# Neurobiological Mechanisms of Action of Transcranial Direct Current Stimulation (tDCS) in the Treatment of Substance Use Disorders (SUDs)—A Review

**DOI:** 10.3390/jcm14144899

**Published:** 2025-07-10

**Authors:** James Chmiel, Donata Kurpas

**Affiliations:** 1 Faculty of Physical Culture and Health, Institute of Physical Culture Sciences, University of Szczecin, Al. Piastów 40B blok 6, 71-065 Szczecin, Poland; 2 Division of Research Methodology, Department of Nursing, Faculty of Nursing and Midwifery, Wrocław Medical University, 51-618 Wrocław, Poland

**Keywords:** transcranial direct current stimulation, tDCS, non-invasive brain stimulation, neuromodulation, neurostimulation, SUD, substance use disorder, alcohol, methamphetamine, cocaine, nicotine, EEG, electroencephalography, electroencephalogram, brain oscillations, QEEG, fMRI, neurophysiology, neural correlates, neural imaging

## Abstract

**Introduction:** Substance use disorders (SUDs) pose a significant public health challenge, with current treatments often exhibiting limited effectiveness and high relapse rates. Transcranial direct current stimulation (tDCS), a noninvasive neuromodulation technique that delivers low-intensity direct current via scalp electrodes, has shown promise in various psychiatric and neurological conditions. In SUDs, tDCS may help to modulate key neurocircuits involved in craving, executive control, and reward processing, potentially mitigating compulsive drug use. However, the precise neurobiological mechanisms by which tDCS exerts its therapeutic effects in SUDs remain only partly understood. This review addresses that gap by synthesizing evidence from clinical studies that used neuroimaging (fMRI, fNIRS, EEG) and blood-based biomarkers to elucidate tDCS’s mechanisms in treating SUDs. **Methods:** A targeted literature search identified articles published between 2008 and 2024 investigating tDCS interventions in alcohol, nicotine, opioid, and stimulant use disorders, focusing specifically on physiological and neurobiological assessments rather than purely behavioral outcomes. Studies were included if they employed either neuroimaging (fMRI, fNIRS, EEG) or blood tests (neurotrophic and neuroinflammatory markers) to investigate changes induced by single- or multi-session tDCS. Two reviewers screened titles/abstracts, conducted full-text assessments, and extracted key data on participant characteristics, tDCS protocols, neurobiological measures, and clinical outcomes. **Results:** Twenty-seven studies met the inclusion criteria. Across fMRI studies, tDCS—especially targeting the dorsolateral prefrontal cortex—consistently modulated large-scale network activity and connectivity in the default mode, salience, and executive control networks. Many of these changes correlated with subjective craving, attentional bias, or extended time to relapse. EEG-based investigations found that tDCS can alter event-related potentials (e.g., P3, N2, LPP) linked to inhibitory control and salience processing, often preceding or accompanying changes in craving. One fNIRS study revealed enhanced connectivity in prefrontal regions under active tDCS. At the same time, two blood-based investigations reported the partial normalization of neurotrophic (BDNF) and proinflammatory markers (TNF-α, IL-6) in participants receiving tDCS. Multi-session protocols were more apt to drive clinically meaningful neuroplastic changes than single-session interventions. **Conclusions:** Although significant questions remain regarding optimal stimulation parameters, sample heterogeneity, and the translation of acute neural shifts into lasting behavioral benefits, this research confirms that tDCS can induce detectable neurobiological effects in SUD populations. By reshaping activity across prefrontal and reward-related circuits, modulating electrophysiological indices, and altering relevant biomarkers, tDCS holds promise as a viable, mechanism-based adjunctive therapy for SUDs. Rigorous, large-scale studies with longer follow-up durations and attention to individual differences will be essential to establish how best to harness these neuromodulatory effects for durable clinical outcomes.

## 1. Introduction

Substance use disorders (SUDs) are characterized by recurrent use of psychoactive substances that severely impair health or daily functioning, or generate pronounced distress. From a clinical perspective, the primary frameworks for diagnosing SUDs are the World Health Organization’s International Classification of Diseases, 11th Revision (ICD-11), and the American Psychiatric Association’s Diagnostic and Statistical Manual of Mental Disorders, Fifth Edition (DSM-5). In the ICD-11, an important distinction is made between harmful or patterned substance use and substance dependence—where impaired control, prioritization of drug use over other activities, and continued use despite adverse outcomes are the defining features [1]. In contrast, the DSM-5 merges the earlier “abuse” and “dependence” categories into a single spectrum of SUD, determined by 11 diagnostic criteria. Meeting two or more of these criteria indicates SUD, with mild, moderate, or severe severity levels based on the number of symptoms. This change in the DSM-5 highlights that addiction can range from relatively mild to a chronic, compulsive disorder rather than being an all-or-nothing condition [2].

On a global scale, SUDs are widespread and represent a substantial public health burden [3]. International surveys consistently show that nicotine and alcohol use disorders are pervasive: around 17% of adults meet the criteria for nicotine (tobacco) use disorder annually [4], and about 8.6% meet the criteria for alcohol use disorder [5]. Although disorders involving opioids, cannabis, and stimulants have a somewhat lower overall prevalence, they still affect tens of millions of people worldwide [6]. Men generally display higher rates of SUD—approximately 1.5 to 2 times more excellent—than women across most regions [7]. The overall disease burden is immense; in 2017 alone, about 30 million years lived with disability were attributed to SUDs [8]. These disorders are also a major contributor to preventable deaths globally, with tobacco and alcohol ranking among the leading risk factors for premature mortality [9,10]. Tobacco use, for instance, was estimated to cause 8.71 million deaths worldwide in 2019 [11].

Characteristic symptoms and progression patterns commonly appear among individuals with SUD. Those affected often report intense cravings and a loss of control over substance use, such as using more than intended or struggling to cut back [12]. As SUD worsens, substance use begins to dominate daily activities, overshadowing responsibilities in personal [13], occupational [14], or educational spheres [15]. Tolerance (the need for larger quantities to achieve the same effect) and withdrawal (physical or psychological symptoms upon reducing or ceasing use) signal the neuroadaptations arising from repeated use [16]. A key diagnostic hallmark is continued substance use despite serious consequences, including medical, legal, or relational problems. SUDs typically unfold in a relapsing–remitting pattern: in the early stages (mild SUD), some degree of control is preserved, yet continued misuse may escalate to a more entrenched, compulsive form. Coexisting psychiatric conditions—such as depression [17], anxiety disorders [18], bipolar disorder [19], or post-traumatic stress disorder [20]—are prevalent in those with SUDs and can worsen both the course of illness and treatment outcomes. These secondary mental health problems can initiate substance use (as a coping strategy) and, in turn, be aggravated by persistent misuse, creating a self-reinforcing cycle [21].

Without proper intervention, SUDs can have dire and widespread effects on both individual well-being and society. Chronic misuse harms numerous organ systems: prolonged alcohol consumption, for example, often leads to liver cirrhosis [22] and heart disease [23]; tobacco use is strongly linked to cancer [24] and pulmonary illnesses [25]; and injecting drugs can introduce blood-borne infections like HIV [26] or hepatitis [27]. SUDs also significantly contribute to mortality through overdoses [28], accidents [29], and violence [30]. In 2018, the United States alone witnessed over 67,367 overdose deaths, most associated with opioids, and globally, millions of fatalities each year are tied to substance use, including alcohol and tobacco [28]. The social ramifications of SUD are similarly serious, fueling family discord [11], child neglect [31], and criminal activity [32]. In purely economic terms, the impact is enormous: in the U.S., healthcare expenses, productivity losses, and criminal justice costs stemming from substance misuse surpass USD 442 billion yearly [33]. Individuals with severe SUD may face joblessness [14], financial difficulties [34], and even homelessness [35].

The neurobiology of SUDs is complex. Addictive substances exert their powerful appeal by co-opting the brain’s reward system, mainly through an acute increase in dopamine levels within the mesolimbic pathway, particularly in the ventral tegmental area and nucleus accumbens. These dopamine spikes reinforce the likelihood of repeated use. Over time, repeated exposure leads to significant neuroadaptive changes in the reward circuitry and stress and executive function networks. The result is a progression from intentional or sporadic use to compulsive patterns: the reward circuitry becomes less responsive to non-drug stimuli but more reactive to drug cues, the extended amygdala (the brain’s stress center) becomes overly active, and prefrontal areas essential for self-control are compromised. Consequently, affected individuals experience heightened craving, reduced enjoyment from previously rewarding activities, and diminished inhibition regarding drug-seeking behavior [36,37,38].

Even though they are multifaceted, SUDs are treatable, and a range of evidence-based interventions can substantially enhance outcomes. Often termed medication-assisted treatment (MAT) coupled with psychosocial support, the most effective strategies integrate both pharmaceutical and behavioral components [39]. In terms of pharmacotherapy, methadone or buprenorphine is used to treat opioid disorders [40]; varenicline or nicotine replacement therapy can address tobacco dependence [41]; and naltrexone or acamprosate may help with alcohol misuse [42]. These medications alleviate cravings, reduce withdrawal symptoms, or dampen the substance’s reward effects. On the behavioral side, treatments such as contingency management, motivational interviewing, and cognitive behavioral therapy target the psychological and behavioral aspects of addiction by imparting tools to resist cravings, modify maladaptive thoughts, and reinforce healthier behaviors [43,44,45]. Because SUD often follows a chronic, relapsing trajectory, a chronic care model is recommended, ensuring a continuous, adaptive treatment that addresses co-occurring physical or mental health issues and normalizes relapse as an indication for treatment adjustment rather than failure. A multidisciplinary approach—combining medication, therapy, social support, and consistent follow-up—yields the most favorable prospects for sustained recovery.

SUDs are a severe burden on the health and life of patients. Therefore, it is necessary to develop effective and safe treatment techniques that directly target the brain because we know that this group of disorders is characterized by altered brain function. One such technique may be transcranial direct current stimulation (tDCS), a technique of electrical brain stimulation from the group of noninvasive brain stimulation (NIBS), which has been intensively studied for two decades in terms of its effectiveness in treating many psychiatric and neurological diseases.

tDCS delivers a low-intensity direct current (usually ranging from 0.5 to 2 mA) through at least two scalp electrodes—an anode (positively charged) and a cathode (negatively charged) [46]. A portion of this current penetrates the skull to modulate neuronal resting membrane potentials, influencing cortical excitability in a polarity-dependent manner [47,48]. Anodal stimulation typically depolarizes neurons, increasing their likelihood of firing, while cathodal stimulation hyperpolarizes neurons and reduces excitability [49]. Although tDCS is inherently diffuse, altering electrode size or placement can help localize stimulation to smaller areas [50], and standard protocols often last from 5 to 30 min with typical intensities of 1–2 mA.

Mechanistically, tDCS engages both short- and long-term processes involving plasticity-related brain changes. The immediate or short-term effect emerges from subthreshold depolarization or hyperpolarization of neuronal membranes [51], which can persist for several minutes after a single session. Longer sessions—particularly those exceeding 10 min at 1–2 mA—can produce lasting changes in cortical excitability that continue for at least an hour [47]. Repeated stimulation further enhances these effects, partly through calcium-dependent synaptic plasticity mechanisms mediated by NMDA receptors in glutamatergic neurons and concurrent modulation of GABAergic inhibition [52,53,54,55,56]. Additionally, tDCS can affect neurotransmitter systems, including norepinephrine [57] and dopamine [58].

While anodal stimulation is broadly thought to increase cortical excitability and cathodal stimulation to decrease it [59], evidence indicates that the actual effect of cathodal stimulation can vary depending on factors like stimulation intensity, duration, and neuronal orientation [60,61,62]. By altering neuronal plasticity locally and in connected brain networks, tDCS has shown promise for treating a range of clinical conditions with minimal side effects, typically limited to mild scalp tingling or itching [63].

There have been many published reviews of tDCS in SUDs [64,65,66,67,68,69,70,71,72,73,74,75,76,77,78,79,80]. tDCS has been shown to reduce the craving and frequency of substance use. For example, tDCS can be helpful in patients with a long history of smoking in terms of cessation and abstinence rates [60]. tDCS also improves the symptoms of opioid addiction [67]. tDCS may also be helpful in alleviating cognitive dysfunction in SUDs [65]. tDCS also reduces cravings in alcohol dependence [70]. There is also ample evidence that it can reduce relapse rates [71]. In most cases, stimulation was applied to the left or right dorsolateral prefrontal cortex (DLPFC). Various protocols have been used, from a single session to treatment protocols lasting up to 10 sessions over 2 weeks. Researchers in review papers mention that knowledge about the mechanisms of action of tDCS in the treatment of addictions is limited, despite the frequency of its use. The number of these reviews indicates that this is an emerging area of research. However, none of the reviews have thoroughly investigated the mechanisms of action of tDCS in SUDs. Knowledge of these mechanisms is essential for further research in this area, improving existing protocols, and improving clinical efficacy. This mechanistic review aims to fill this gap and discuss the potential mechanisms of action of tDCS in SUDs by searching for studies that have tested tDCS in this area using non-behavioral tools such as neuroimaging techniques and blood tests. The results obtained are supplemented with a broader interpretation derived from several neurobiological and neuroimaging studies. We catalog the neurocircuits, electrophysiological indices and blood-borne biomarkers reported to change after single- and multi-session tDCS, identify points of convergence and divergence in these mechanistic signatures as a function of stimulation montage, current density, session number, and withdrawal state, and evaluate the extent to which the observed biological shifts mediate proximal clinical outcomes such as craving, inhibitory control performance and time-to-relapse. By exposing recurring methodological gaps—small samples, heterogeneous protocols, and brief follow-ups—and synthesizing multimodal evidence from fMRI, fNIRS, EEG/ERP and neuro-immune measures, this review provides a consolidated mechanistic framework and a set of testable hypotheses to guide parameter optimization and next-generation randomized trials of tDCS as an adjunctive therapy for substance-use disorders.

## 2. Methods

This mechanistic review aims to thoroughly assess tDCS’s mechanisms of action in treating SUDs. A thorough literature search and stringent selection criteria were used to guarantee the reliability and applicability of the presented evidence. Finding case studies and clinical trials that assess the use of neuroimaging technologies and other non-behavioral diagnostic tools was the focus of the research, which followed the accepted standards for systematic reviews and evidence synthesis (PRISMA) [81]. Note that this review does not follow all aspects of the PRISMA methodology commonly employed in systematic reviews and instead takes a mechanistic rather than a fully systematic approach.

### 2.1. Data Sources and Search Strategy

J.Ch. and D.K. used a combination of specific terms in an independent, standards-compliant Internet search to gather this evaluation. “Transcranial direct current stimulation”, or “tDCS”, and “alcohol”, “drinking”, “alcohol”, “craving”, “substance”, “substance use disorder”, “SUD”, “nicotine”, “cigarettes”, “tobacco”, “smoking”, “methamphetamine”, “meth”, “cocaine”, “crack”, “heroin”, “methadone”, “cannabis”, and “marijuana” were among them. In December 2024, a thorough search was conducted in several databases, including PubMed/Medline, Research Gate, Google Scholar, and Cochrane, emphasizing articles released between January 2008 and April 2025.

### 2.2. Study Selection Criteria

Studies had to be clinical trials or case studies published in English between 2008 and 2025 to be eligible for inclusion. They had to use neuroimaging and other non-behavioral measurement methods to investigate how tDCS affected SUD patients. Non-English-language publications and review papers were not taken into account.

### 2.3. Screening Process

A multi-step screening procedure was used to guarantee the inclusion of pertinent research and the exclusion of studies that did not fit the predetermined criteria. Two independent reviewers—J.Ch. and D. K.—reviewed abstracts and titles during the first screening process.

#### 2.3.1. Title and Abstract Screening

Each reviewer assessed the independently available records’ abstracts and titles throughout the first screening to see if they satisfied the inclusion requirements. Studies examining the impact of tDCS on SUD patients utilizing neuroimaging and other non-behavioral measuring techniques were the main focus.

#### 2.3.2. Full-Text Assessment

The chosen papers underwent a thorough full-text assessment after the initial screening. To ensure that the studies were clinical trials or case studies published in English between January 2008 and April 2025, the reviewers carefully examined each publication to verify eligibility.

## 3. Results

**Figure 1:**
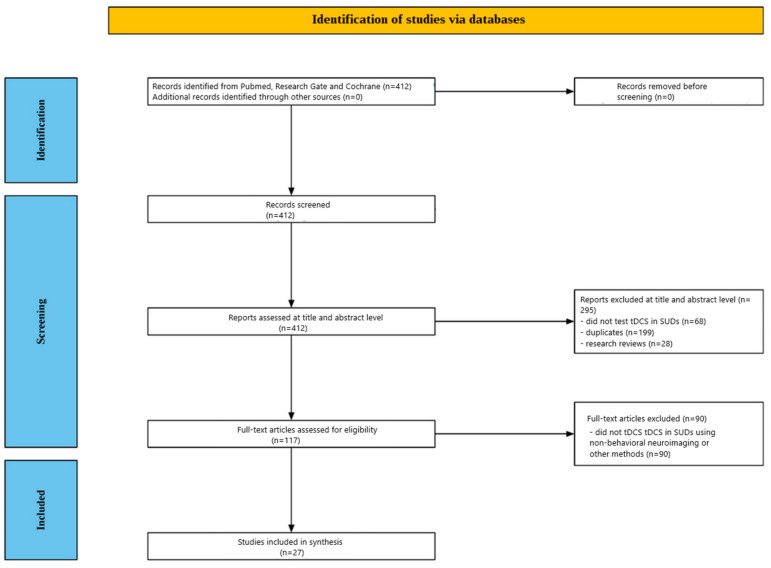
Flow chart depicting the different phases of the systematic review.

Figure 1 provides a summary of the screening process. Following a review of the titles and abstracts, 295 publications were eliminated from the 412 studies that were first found through database searches. Further, 28 review articles, 199 duplicates, and 68 studies that did not assess tDCS in SUD were among the reasons for exclusion. Additionally, 90 articles were later eliminated for not examining the effect of tDCS on patients with SUD using neuroimaging and other non-behavioral measuring modalities after the remaining 117 papers underwent a comprehensive full-text evaluation. In the end, twenty-seven (27) articles were included in this evaluation after meeting the inclusion criteria.

The included studies [82,83,84,85,86,87,88,89,90,91,92,93,94,95,96,97,98,99,100,101,102,103,104,105,106,107,108] were published between 2012 and 2023. Studies [82,83,84,85,86,87,88,89,90,91,92,93] used functional magnetic resonance imaging, studies [87,94,95,96,97,98,99,100,101,102,103,104,105] used various forms of electroencephalography, study [106] used functional near-infrared spectroscopy (fNIRS), and studies [107,108] used blood measurements.

### 3.1. Summary of Included Studies

#### 3.1.1. fMRI Studies

The fMRI results are presented in Table 1.

##### Nicotine-Related Studies

Four investigations—three single-session cross-over experiments and one five-day course—examined whether prefrontal tDCS alters craving, cognitive performance and brain activity in people who smoke. Together they involved 122 smokers (27% women) and 28 non-smokers; stimulation intensity ranged from 1 mA [84] to 2 mA [82,83,85].

##### Behavioral Outcomes

Study [82] contrasted smokers tested sated (nicotine patch) or after 12 h abstinence (placebo patch) with 28 matched non-smokers during three counter-balanced tDCS conditions (anode-left/cathode-right, polarity-reversed, sham). Task accuracy and speed were dictated by nicotine state, not stimulation: abstinence slowed responses across all paradigms (*p* < 0.05) and lowered N-back accuracy (*p* = 0.003) as well as Faces task accuracy (*p* = 0.02). No tDCS main or interaction effect reached significance for any behavioral variable. Study [83] delivered ten 20 min sessions (anode midway F4/Fp2) over five days in 29 smokers who wished to quit (active = 14, sham = 15). Daily cigarette count fell across the treatment week (linear trend *p* < 0.001) but the slope was identical in both groups (*p* = 0.708). Session-by-session craving ratings, however, dropped more steeply under active stimulation (group main effect: F_1,25_ = 5.16, *p* = 0.031, η^2^ = 0.16). Study [84] used a counter-balanced within-subject design (left anode, 1 mA, 30 min) for 32 male smokers. Cue-induced craving rose overall (t_63_ = 2.37, *p* = 0.021), but the increase was significantly smaller after real tDCS (t_31_ = −2.32, *p* = 0.027, *d* = 0.41). Reaction time and accuracy on the cue task were unchanged. Study [85] randomized 46 male smokers to anodal left, anodal right or sham (2 mA, 20 min) stimulation while they performed a go/no-go paradigm. Only the right-anode montage shortened go-trial reaction time compared with sham (*p* = 0.008); commission and omission errors were unaffected.

##### Task-Evoked BOLD Responses

Intra-task contrasts revealed montage-specific modulation of prefrontal and cingulo-temporal regions. In error monitoring (Parametric Flanker [82]), a Group × tDCS interaction emerged in the right anterior cingulate cortex (cluster-wise p_corr = 0.03): anode-left stimulation increased ACC activity more than both reversed polarity and sham, with the largest effect in sated smokers (*p* = 0.005 vs. sham). In smoking cue reactivity [84], a 2 × 2 ANOVA showed no main tDCS effect, but the tDCS × Cue interaction was significant in the left superior frontal gyrus (t_31_ = 3.81, *p* = 0.0006) and middle frontal gyrus (t_31_ = 2.72, *p* = 0.011): real tDCS suppressed smoking-minus-neutral activation. In five-day cue reactivity [83], flexible factorial analysis isolated a single cluster in the right dorsal posterior cingulate cortex, showing a Time × Group interaction (*p* < 0.005, k ≥ 85): active tDCS increased dPCC reactivity (t = −2.71, *p* = 0.018) whereas sham decreased it (t = 3.15, *p* = 0.012). In motor inhibition [85], anodal left tDCS reduced BOLD during go trials in the left precuneus, middle occipital gyrus and cerebellar crus I (all p_corr < 0.05); anode-right produced similar decreases in the right precuneus and left middle occipital gyrus. Between-group contrasts revealed additional thalamic, lingual and parahippocampal differences.

##### Large-Scale Network Effects

In [82], during 3-back > 0-back, nine DMN nodes (e.g., hippocampus, precuneus) were more strongly de-activated under anode-left tDCS than under either reversed polarity or sham (α_corr < 0.01). The effect disappeared when smokers were acutely abstinent. In [84], psychophysiological interaction analysis revealed that real tDCS inverted the coupling between the stimulated left DLPFC (seed) and right parahippocampal gyrus (t_31_ = 3.86, *p* = 0.0005); greater decoupling correlated with larger craving reductions (r = −0.52, *p* = 0.002). Resting-state scans showed a parallel increase in left DLPFC ↔ parahippocampal connectivity (t_31_ = 3.33, *p* = 0.002). In [85], post-stimulation seed-based connectivity demonstrated bidirectional changes: anode-left stimulation heightened left DLPFC connectivity with right parahippocampal gyrus but lowered links to cerebellum and thalamus; anode-right stimulation produced the mirror image. During no-go trials, anode-right stimulation strengthened right DLPFC→cerebellum coupling and weakened links to left caudate and temporal pole.

##### Alcohol-Related Studies

Five datasets, each using five consecutive daily sessions of 2 mA bilateral DLPFC tDCS, investigated neural and clinical responses in recently detoxified in-patients with alcohol (or combined alcohol/crack-cocaine) dependence. Collectively, these experiments enrolled 122 men (mean age range ≈ 24–57 year) and employed pre-/post-course MRI to quantify functional and structural plasticity.

##### Behavioral and Clinical Outcomes

In [86], time-to-first-drink after discharge doubled in the active arm (median 30 day vs. 15 day; Breslow-Wilcoxon χ^2^ = 6.2, *p* = 0.013). Faster stop-signal reaction time gains co-varied with network reorganization (*r* = 0.57, *p* = 0.014). Study [87] showed that across five sessions, craving for crack-cocaine (but not alcohol, data not separated) fell in proportion to diffusion-tensor improvements in the left vmPFC→NAcc tract (∆FA *R*^2^ = 0.34, ∆ADC *R*^2^ = 0.29; *p* < 0.05). In [88], after treatment, individuals showing the largest DLPFC BOLD increase during alcohol cues remained abstinent longest (Spearman ρ = 0.426, *p* = 0.048). In [89], at four-month follow-up, the relapse probability was 19% under active vs. 38% under sham (χ^2^ = 3.92, *p* = 0.048) stimulation; among women, the rates were 9.1% vs. 50%. Directed connectivity gain from left DLPFC to the incentive salience network predicted continued abstinence (*p* = 0.032).

##### Task-Evoked BOLD Responses

In [88], a VICE cue reactivity paradigm showed no baseline group differences. After five sessions, the active group exhibited significantly greater bilateral DLPFC activation (Post–Pre) than sham (cluster-level FWE-corrected). In [89], no direct cue task was used, but causal discovery of resting data identified strengthened left-DLPFC output toward incentive salience and negative emotionality circuits uniquely in the active arm.

##### Resting-State Network Reorganization

In [86], across sparsity thresholds, global efficiency rose (F_1,19_ = 8.97, *p* = 0.007) and global clustering fell (F_1,19_ = 8.75, *p* = 0.008) after active—but not sham—stimulation. Network-based statistics (T = 4.5, 5000 permutations) isolated a right-lateralized fronto-cingulo-motor sub-network (right MFG, right ACC, right SMA, left mid-cingulate) whose connectivity increased only in the active group. Causal discovery analysis in [89] revealed bidirectional effects: active tDCS increased left-DLPFC-to-network edges for incentive salience and negative emotionality, whereas sham decreased them; no montage effect emerged for executive-go or executive-stop pathways.

##### Structural Plasticity (Diffusion-Tensor Imaging)

In [87], among three pre-registered tracts, only left vmPFC→NAcc changed. Group × Time interactions were significant for voxel count (F_1,11_ = 7.4, *p* = 0.020), fractional anisotropy (F_1,11_ = 8.1, *p* = 0.016), and apparent diffusion coefficient (F_1,11_ = 5.99, *p* = 0.032; partial η^2^ ≈ 0.35–0.42). Post hoc contrasts confirmed higher post-treatment voxel count (*p* = 0.049 vs. baseline, *p* = 0.020 vs. sham) and FA (*p* = 0.018 vs. baseline, *p* = 0.016 vs. sham) in the active group.

##### Methamphetamine-Related Studies

Four publications explored the neural impact of a single 20 min, 2 mA prefrontal tDCS session in early-abstinent men with methamphetamine use disorder, yielding data from 105 participants in total (all male). Two trials used a double-blind crossover design with the anode on F4 and cathode on F3 [90,92], while a larger parallel-group study and its subsequent dose–response re-analysis placed the anode on F4 and the cathode over the contralateral supra-orbital region [91,93].

##### Behavioral Effects

Across the two crossover experiments, active stimulation reliably reduced craving. In [90], the mean self-reported urge fell by roughly 15 points (on a 0–100 scale) after real tDCS, but by only about 1 point after sham (*p* = 0.03). Study [92] replicated this pattern, again showing a significantly larger active-minus-sham drop. By contrast, in the 60-person parallel trial [91], both groups reported similar post-scan relief, and the later electric field analysis [93] suggested that craving change was better predicted by each participant’s baseline frontoparietal connectivity and delivered field strength than by group assignment itself. No study found stimulation-linked changes in reaction-time or accuracy measures.

##### Task-Evoked BOLD Modulation

Study [91] showed that cue-evoked activity evolved in opposite directions across arms: sham subjects displayed the expected habituation between the first and second drug image block, whereas active tDCS increased the recruitment of five left-hemisphere clusters—the middle frontal gyrus, anterior insula, inferior parietal lobule, precuneus and inferior frontal gyrus (cluster-wise *p* < 0.005). Study [92], using generalized psychophysiological interaction (gPPI), found that stimulation strengthened connectivity from an executive control prefrontal seed to a large visual/precuneus cluster and, conversely, weakened connectivity from a default-mode seed to a parietal cluster—changes that moved the network balance toward externally oriented control processes.

##### Resting-State Network Reorganization

Immediately after real stimulation in [90], posterior default-mode connectivity (posterior cingulate, precuneus, middle temporal gyrus) fell, while coupling within the executive control network and salience network rose; the magnitude of DMN downregulation and ECN/SN upregulation correlated with the size of each participant’s craving reduction. Study [92] extended these findings by showing enhanced integration within, and between, the ECN and ventral attention network, together with reduced intra-DMN and DMN-to-attention coupling. In study [91], a seed placed in the right superior frontal gyrus (the site receiving the strongest simulated current) exhibited a time-by-group interaction: seed-to-parietal connectivity decreased only after active tDCS, while increasing after sham.

##### Electric Field Dose–Response Relationships

Voxel-, ROI- and cluster-level analyses in the 60-subject dataset found no direct correlation between electric field (EF) intensity and local BOLD change, yet a network-scale association emerged. Stronger right-frontal EF—inferred from individualized head models—predicted a larger increase in frontoparietal functional connectivity during cue exposure. Exploratory work further indicated that participants with both high baseline frontoparietal coherence and higher EF experienced the lowest subsequent cue-induced craving [93]. Across the included functional MRI (fMRI) investigations [82,83,84,85,86,87,88,89,90,91,92,93], tDCS targeting prefrontal areas—most often the dorsolateral prefrontal cortex (DLPFC)—consistently modulated neural networks linked to craving, executive function, and salience. While individual studies varied widely in design (substance of abuse, single- or multi-session protocols, and cognitive/behavioral tasks), three overarching findings emerged. The fMRI results are presented in Table 1. First, large-scale network activity and connectivity changes appeared across both single- and repeated-session designs [82,84,85,86,87,90,92]. Default-mode network (DMN) alterations often manifested as increased deactivation or reduced connectivity in regions such as the precuneus or posterior cingulate cortex [82,90,92], potentially reflecting a dampening of cue-related ruminative processes. In parallel, the executive control network (ECN) showed enhanced activation or connectivity, notably when the DLPFC was the anode [82,84,86,90]. Reward- and salience-related circuits, including the anterior cingulate cortex (ACC), insula, and ventral striatum, also exhibited notable shifts; for example, repeated bilateral DLPFC stimulation correlated with white matter changes in the vmPFC–nucleus accumbens pathway [87], and single-session tDCS modulated cingulate or insular responses to drug cues in some stimulant use studies [90,92].

#### 3.1.2. EEG Studies

The EEG results are presented in Table 2. A central theme across these studies is that tDCS can alter ERP indices (e.g., P3, N2, LPP, ERN) related to attention, inhibition, and error processing—even when overt behavioral measures show limited or delayed improvements. For instance, in study [94] with habitual tobacco smokers, the absence of short-term changes in accuracy, reaction times, or early ERP components (N2, P3, ERN) contrasted with a subsequent three-month improvement in reaction times and a relative decrease in P3 amplitude to smoking-related cues. This time lag suggests that neural modifications induced by tDCS might not always translate into immediate behavioral change but may facilitate longer-term adaptations in inhibitory control and reactivity to drug cues.

Similarly, a study [95] on hazardous drinkers engaging in approach bias retraining found no direct tDCS effect on approach bias, craving, or alcohol consumption in the short term. However, EEG recordings during an oddball or cue reactivity task indicated that active tDCS was associated with a more significant reduction in craving ratings for alcohol pictures—a finding that purely behavioral measures might have overlooked. These patterns echo the broader observation that electrophysiological changes (e.g., P300 amplitude modulations) sometimes precede and predict eventual behavioral and clinical outcomes.

Several studies targeting alcohol dependence (e.g., [87,96,97]) show similarly mixed clinical and electrophysiological effects. For example, study [96] found that actual tDCS participants exhibited more significant reductions in craving and depression symptoms than sham-treated individuals, yet they paradoxically had higher relapse rates. Meanwhile, EEG/ERP measures suggested reduced prefrontal cortex activation changes in the actual tDCS group, raising questions about how neural modulation interacts with other psychosocial or clinical factors determining relapse.

In study [97], more substantial ERP changes (specifically, P3 amplitude enhancements following alcohol-related sounds) were seen mainly in Lesch Type IV alcohol-dependent participants, suggesting that individual differences in SUD subtype may modulate the impact of tDCS. The finding that Type IV individuals also showed improved executive functioning (Frontal Assessment Battery scores) underscores the possibility that tDCS benefits are not uniform but may be selective for specific patient subgroups, potentially those with pronounced frontal executive deficits.

Investigations involving cocaine/crack-cocaine users (e.g., [87,102,103,104]) further highlight how repeated or even single sessions of bilateral DLPFC tDCS can suppress hyperactivation in frontal regions (e.g., ACC, left DLPFC) during drug cue exposure and enhance activation in alternative, potentially more adaptive cortical networks (e.g., vmPFC, orbitofrontal cortex). In these patients, ERPs in the P3 or N2 time window often point to a shift in salience assigned to drug cues versus neutral stimuli, again illustrating that tDCS may facilitate a rebalancing of attention or reductions in cue-provoked motivational responses.

In samples with amphetamines (e.g., [100]), real tDCS reduced reaction time costs (invalid minus valid cues) and increased P300 amplitudes in neutral-cue conditions, suggesting improved attentional control and a possible normalization of cortical processing. Meanwhile, in opioid use disorder populations, study [101] reported that both left-anode/right-cathode and right-anode/left-cathode tDCS led to decreases in slow-wave amplitude (delta, theta, alpha) and increases in functional connectivity (coherence), consistent with a broad reorganization of cortical activity.

Finally, studies examining binge drinkers or other subgroups show that the effects of tDCS on behavioral measures are not always robust or consistent. Yet, ERP components such as N2, P3 or LPP often exhibit greater sensitivity to the stimulation [98,99,105]. For example, study [99] noted that binge drinkers and non-binge drinkers did not differ behaviorally following tDCS. Still, their N2 or P3 components diverged, suggesting that pre-existing neural patterns (e.g., greater impulsivity, altered conflict monitoring) could interact with the neuromodulatory effects of tDCS.

#### 3.1.3. fNIRS Studies

The fNIRS results are presented in Table 3. Study [106] investigated whether tDCS would alter craving, heart-rate variability (HRV), and prefrontal hemodynamics in adult smokers when they were exposed to smoking-related cues. Twenty-nine university students who reported smoking at least weekly were randomly assigned to receive real or sham tDCS during a structured, 20 min “in vivo” smoking-cue exposure session designed to elicit craving. The tDCS protocol used a 2 mA current for 15 min, placing the anode over the left dorsolateral prefrontal cortex (F3) and the cathode over the orbitofrontal region (Fp2), while a sham mode only mimicked the ramp-up without sustaining current. Self-reported ratings assessed craving, HRV was derived from electrocardiogram data (focusing on low-frequency, high-frequency, and the ratio between them), and prefrontal hemodynamics were recorded with functional near-infrared spectroscopy (fNIRS) to measure concentration changes in deoxygenated hemoglobin in orbitofrontal and dorsolateral prefrontal cortex regions. Repeated-measures ANOVAs indicated that subjective craving rose significantly across the 20 min smoking-cue exposure (time effect: F(3,78) = 29.65, *p* < 0.001, η^2^ = 0.091), but there was no time-by-stimulation interaction (F(3,78) = 0.80, *p* = 0.514). Heart-rate variability likewise increased in the low-frequency domain over time (F(3,69) = 5.87, *p* = 0.001, η^2^ = 0.20), without a significant interaction by tDCS condition (F(3,69) = 1.64, *p* = 0.187). Regarding prefrontal hemodynamics, left BA9 showed a time-by-stimulation effect (F(2,48) = 3.26, *p* = 0.039, η^2^ = 0.140), and seed-based correlation analyses revealed that connectivity between the orbitofrontal cortex channel (CH48) and the dorsolateral prefrontal region (CH6) was higher in the real tDCS group compared to sham (effect size *d* = 0.66, *p* < 0.001). No group differences emerged between the high-frequency heart-rate component and the low-/high-frequency ratio.

#### 3.1.4. Studies Using Blood Tests

The results from the blood tests are presented in Table 4. Study [107] compared the effects of different tDCS protocols on serum levels of brain-derived neurotrophic factor (BDNF) and on various psychological measures in individuals with opioid addiction. Thirty male patients with opioid use disorder were divided into three groups of ten, each receiving either left-anodal/right-cathodal DLPFC stimulation, right-anodal/left-cathodal DLPFC stimulation, or a sham stimulation protocol. All participants were assessed before and after ten sessions of their assigned tDCS protocol. Researchers measured serum BDNF (using ELISA), as well as depression, anxiety, and stress via the Depression Anxiety Stress Scale (DASS) and craving with the Desires for Drug Questionnaire (DDQ). For tDCS, two sponge electrodes delivered a 2 mA current for 20 min per session: the first active group had an anode over left DLPFC (F3) and a cathode over right DLPFC (F4), the second active group had an anode over right DLPFC (F4) and a cathode over left DLPFC (F3), while the sham group received minimal actual stimulation. Statistical comparisons of post-test data showed that, for BDNF, group B differed significantly from group C (*p* = 0.042), while no significant difference emerged between group A and group C. For depression, group A significantly differed from group C (*p* = 0.023), whereas group B did not. With anxiety, there was a significant difference between group A vs. group C (*p* = 0.001) and group B vs. group C (*p* = 0.006). For stress, group A vs. group C showed a significant difference (*p* = 0.014), but group B vs. group C did not. For craving, group A vs. group C (*p* < 0.001) and group B vs. group C (*p* = 0.002) both reached statistical significance. There was no direct difference between the two active protocols (group A vs. group B) on these measures, but each differed from sham on select outcomes.

The randomized, double-blind, sham-controlled clinical trial [108] investigated the effects of bilateral tDCS on craving, tumor necrosis factor-alpha (TNF-a) and interleukin-6 (IL-6) expression levels, and impulsivity in adult males with opioid use disorder (OUD). In detail, 31 men with OUD were assigned to three groups: a left-anode/right-cathode DLPFC stimulation group, a right-anode/left-cathode DLPFC stimulation group, and a sham stimulation group. Each participant underwent 10 consecutive 20 min tDCS sessions (2 mA). The study measured (1) opioid craving using the Desires for Drug Questionnaire, (2) TNF-a and IL-6 expression levels in blood samples using ELISA, and (3) impulsivity via the Barratt Impulsiveness Scale (BIS-11). A one-way ANOVA on pre- vs. postintervention changes in craving found a significant group effect, *F*(2,28) = 14.683, *p* < 0.001, partial eta-squared = 0.625, indicating that the active tDCS groups (left-anode/right-cathode and right-anode/left-cathode) and the sham group differed overall in reduction in craving. However, paired *t*-tests revealed that all three groups had a statistically significant decrease in craving from pre- to postintervention (*p* < 0.01 for both active groups, *p* = 0.005 for the sham group), with large effect sizes for the active tDCS groups (*d* > 2.0) and a more moderate effect size in the sham group (*d* = 0.52). Levels of IL-6 and TNF-α, measured by ELISA, showed no significant overall group differences, *F*(2,28) = 0.980, *p* = 0.38 for IL-6 and *F*(2,28) = 1.381, *p* = 0.26 for TNF-α. Within-group paired *t*-tests revealed that in the right-anode/left-cathode group, IL-6 decreased significantly (pre-post: *t*(10) = 3.367, *p* = 0.007, *d* = 0.75) and TNF-α declined as well (*t*(10) = 3.094, *p* = 0.01, *d* = 0.86). Despite these decreases, between-group comparisons for cytokine changes did not reach overall significance, partly because IL-6 and TNF-α increased in the sham group.

## 4. Discussion

Transcranial direct current stimulation is a technique that has been intensively studied in the context of treating substance use disorders. This is evidenced by many clinical studies devoted to this topic (over 100). Moreover, much effort has been put into studying the neuronal and neurobiological mechanisms of tDCS in SUDs. Modern neuroimaging techniques such as fMRI and fNIRS, and classical methods such as electroencephalogram, as well as studies of biomarkers of neuroinflammation and neuroplasticity from blood allow us to know how tDCS affects patients with SUDs; thanks to this intervention, they experience a reduction in drug craving and a lower frequency of addictive substance consumption. Evidence from the earlier studies shows that tDCS induces numerous changes in the brain at the neuronal level (activity, functional connectivity), bioelectrical (changes in EEG and various ERP components) and in biomarkers of neuroplasticity and neuroinflammation in blood. This confirms that tDCS can induce lasting neuroplasticity, which consists of brain functional reorganization. This results in the behavioral changes observed in studies on addicted patients. The following sections discuss the findings of the different techniques in the included studies in a complementary manner.

### 4.1. fMRI Outcomes

Across the studies reviewed, several overarching patterns and important nuances emerge regarding the capacity of tDCS to modulate neural activity and behavior in SUDs. Although the specific stimulation montages, protocols, and target populations varied substantially, a unifying theme is that prefrontal cortex (PFC)-focused tDCS can induce detectable changes in large-scale networks (particularly the default mode network, salience network, and executive control network), sometimes accompanied by modest behavioral or clinical improvements. Below, we discuss key findings and interpret them in the context of emerging knowledge on neuromodulation in SUDs.

#### 4.1.1. Network-Level Insights from fMRI

A significant outcome across these tDCS studies is the consistent modulation of major large-scale brain networks implicated in addictive behavior—especially the default mode network (DMN), the executive control network (ECN), and the salience or reward-related networks. While stimulation over the dorsolateral prefrontal cortex (DLPFC) influences local cortical excitability, fMRI analyses further reveal that tDCS can shift the functional connectivity and activation patterns among distributed neural circuits essential for regulating craving, attention, and inhibitory control.

##### Default Mode Network (DMN)

The default mode network (DMN) consists of midline cortical structures—such as the posterior cingulate cortex/precuneus and the medial prefrontal cortex—and lateral parietal as well as medial temporal lobe areas (including the hippocampus and parahippocampal gyrus [109]. Typically, these regions are more active at rest and deactivate during goal-directed tasks [110]. Still, in SUDs, hyperconnectivity or insufficient deactivation of the DMN has been associated with persistent internally focused thinking [111], excessive rumination on drug cues [112], and a higher risk of relapse [113]. In study [82] on nicotine dependence, anodal stimulation of the left dorsolateral prefrontal cortex (DLPFC) prompted more substantial DMN deactivation during a high-load N-back task (3-back minus 0-back). Specifically, nine DMN-linked regions—including the hippocampus, precuneus, and middle cingulate cortex—showed more pronounced deactivation under anodal-left DLPFC (An-dlPFC) relative to reversed polarity or sham. These effects were seen in the “Trait-Smoking” model (comparing sated-smokers to nonsmokers), suggesting that boosting DLPFC excitability can counteract the DMN dysregulation characteristic of chronic smokers by facilitating task-related DMN suppression. Moreover, in the same study’s “Nicotine-State” model, a tDCS × Patch interaction indicated that more substantial DMN deactivation under An-dlPFC occurred when participants were nicotine-sated rather than abstinent, highlighting the potential importance of internal physiological states for how effectively tDCS can modulate DMN activity.

Comparable DMN findings emerged in stimulant-focused studies, particularly in methamphetamine dependence [90,92], where anodal tDCS over the right DLPFC reduced resting-state connectivity in posterior DMN regions such as the posterior cingulate cortex, precuneus, and middle temporal gyrus. These decreases correlated with reduced craving, implying that attenuating DMN hyperconnectivity can dampen rumination and drug-related thought processes. In study [92], active tDCS also altered task-based connectivity between a prefrontal DMN node and parietal cortex, reducing DMN–parietal connectivity in the actual stimulation condition, a result consistent with the hypothesis that improved prefrontal control can impose “top–down” regulation on DMN regions and break craving-related cycles. From a clinical perspective, these DMN modifications could help by lessening drug-oriented internal monologs, enhancing attention to external tasks, or counteracting the cue-driven triggers that typically arise from internally focused states. Additionally, in the nicotine study, the difference in DMN responsiveness when smokers were sated versus abstinent underscores how withdrawal or medication status may affect tDCS outcomes, thus suggesting that practical implementation of tDCS-based interventions might benefit from concurrent symptom management. Although direct behavioral correlates—such as significant improvements in performance—were not always observed, the more pronounced DMN downregulation, particularly during demanding tasks, aligns with improved executive control and potentially helps reduce relapse-prone mental states. Overall, these results reinforce the idea that well-designed tDCS protocols can target DMN hyperactivity in SUDs, thereby promoting more adaptive cognition, mitigating cravings, and ultimately contributing to longer-term abstinence.

##### Executive Control Network (ECN)

The executive control network (ECN) encompasses lateral prefrontal and parietal regions—most notably the dorsolateral prefrontal cortex, inferior parietal lobule, and parts of the anterior cingulate cortex [114]—that together facilitate goal-directed behavior [115], working memory [116], and inhibitory control [117]. In the context of SUDs, dysfunction in the ECN can diminish an individual’s capacity to regulate cravings and resist habitual drug seeking [118,119,120]. Chen et al. note that ECN plays an important role in executive control including craving control, and that tDCS can increase intra-network ECN connectivity [76]. In practical terms, this means stimulated subjects may recruit DLPFC–ACC circuits more effectively to suppress impulses. This is consistent with our EEG results: increased N2/P3 amplitudes after tDCS (seen in many studies) index stronger conflict monitoring and inhibition, which we interpret as neurophysiological markers of heightened ECN engagement. Thus, tDCS may relieve the “brake failure” of addiction by enhancing prefrontal top–down control. Across multiple tDCS studies, fMRI measures reveal that stimulating the DLPFC can induce shifts in ECN function, potentially ameliorating some of these deficits. For example, in study [82], although no significant overall tDCS effect emerged on behavioral outcomes, anodal DLPFC stimulation during the high-load N-back task still correlated with enhanced deactivation of default mode regions and preserved or improved working memory performance in sated smokers. This pattern implies that tDCS-induced changes in prefrontal excitability can boost the ECN’s ability to meet higher cognitive demands.

In a related vein, studies [84,85] in chronic smokers further highlight how anodal stimulation of the DLPFC modulates ECN activity during tasks tapping attention and inhibition. In study [84], left DLPFC tDCS drove functional connectivity changes between the stimulated region and temporal–limbic structures, aligning with diminished craving responses. Meanwhile, study [85] used a go/no-go paradigm and found that right- vs. left-sided DLPFC anodal stimulation produced distinct patterns of prefrontal–parietal–cerebellar connectivity, reflecting the hemisphere-specific contributions of the DLPFC to inhibitory processing and executive control.

Multiple-session protocols in alcohol-dependent populations have underscored the longer-term implications of ECN modulation. In study [86], where participants received five consecutive days of active or sham tDCS, the active group demonstrated not only increased global efficiency in functional networks but also more significant improvements in impulse control and a prolonged time to relapse. Such findings dovetail with study [89], in which repeated left DLPFC stimulation enhanced directed connectivity from the DLPFC to two “addiction networks”—incentive salience and negative emotionality— which both intersect with core ECN nodes and appear essential to maintaining abstinence. Similarly, repeated stimulation of the bilateral DLPFC in study [87] correlated with microstructural changes in reward-related pathways, suggesting that shifts in ECN function may be accompanied by more profound structural remodeling in frontostriatal circuits.

In methamphetamine use, studies [90,92] revealed that actual tDCS sessions, targeting the right DLPFC, led to increased connectivity within the ECN (e.g., between frontal and parietal regions) during both resting-state and cue reactivity paradigms, alongside reductions in craving. These results reinforce that frontal stimulation can recalibrate the ECN’s top–down regulatory capacity, thereby mitigating overactive limbic responses to drug-related cues. Overall, this growing body of evidence suggests that strengthening the ECN via DLPFC-focused tDCS may serve as a potent means of countering the impaired inhibitory control and heightened cue reactivity that characterize SUDs.

##### Salience and Reward-Related Networks

The salience network (SN) and reward-related circuits involve brain regions such as the anterior cingulate cortex (ACC), insula, ventromedial prefrontal cortex (vmPFC), and subcortical structures, including the striatum (e.g., nucleus accumbens) and thalamus [121,122,123,124]. In SUDs, hyperresponsiveness within these systems can fuel craving and compulsive drug-seeking, often overshadowing top–down regulatory signals from the executive control network [124,125]. Numerous tDCS studies have focused on dorsolateral prefrontal cortex (DLPFC) stimulation to rebalance this salience–reward axis by augmenting prefrontal control and diminishing the bottom–up drive of drug cues. DLPFC has direct and indirect projections to these regions. For example, increased causal connectivity from DLPFC to incentive salience networks was directly observed by Camchong et al. in AUD patients [89]. Similarly, one would expect that an uptick in DLPFC excitability could drive down limbic over-responsiveness. The tDCS-induced increases in fronto-striatal and fronto-insular connectivity reported in several fMRI studies support this: when DLPFC is more active, it may impose more regulation on subcortical craving centers [126]. This is further suggested by ERP changes: a larger P3 (parietal positivity) to drug cues might indicate that the stimulus is being more heavily evaluated (or filtered) by a primed control system. Overall, we hypothesize that tDCS shifts the excitation/inhibition balance in the prefrontal cortex (altering glutamatergic/GABAergic tone) and that this shift cascades to reward circuits, reducing their aberrant gain.

In study [82], region-of-interest analyses revealed that anodal tDCS over the left DLPFC interacted with smoker status in the right ACC during an error-monitoring (Parametric Flanker) task, with activity increasing more under actual stimulation than with reversed polarity or sham—especially in sated smokers. Because the ACC is a core hub of the SN, involved in detecting salient conflicts and recruiting cognitive resources, this finding implies that enhanced DLPFC excitability can potentiate ACC responsiveness. Similarly, the study [85] demonstrated changes in ACC connectivity following right- or left-sided DLPFC stimulation during a go/no-go task, suggesting that tDCS polarity and hemisphere can selectively affect salience-related gating of inhibitory processes.

Longer-term plasticity within reward pathways has also been documented. In the study [87], repeated bilateral DLPFC stimulation increased fractional anisotropy (FA) in the left vmPFC–nucleus accumbens tract, correlating with reduced craving in crack-cocaine- or alcohol-dependent individuals. These structural changes imply that tDCS can promote or accelerate white matter remodeling in circuits strongly implicated in motivation and reward valuation. Other studies, such as study [88] and study [89] on alcohol dependence, found that multi-session tDCS targeting the DLPFC not only lowered craving and prolonged abstinence but also produced detectable changes in frontal–subcortical connectivity and activation patterns, reinforcing the notion that salience and reward system modulation is a key mechanism in sustaining recovery.

In stimulant use disorders, studies [90,92] noted that real tDCS to the right DLPFC decreased cue-elicited activity or connectivity in salience processing hubs—including the ACC, posterior cingulate, and insula—while increasing executive control network connectivity. These dual changes were closely linked to concurrent drops in self-reported craving, supporting a model in which upregulating prefrontal function constrains overactive salience circuits that would otherwise prioritize drug cues. Although the study [91] did not observe a reduction in subjective craving relative to sham, the active group exhibited increased activation in left-hemisphere regions (middle frontal gyrus, anterior insula, inferior parietal lobule, and precuneus) and altered frontoparietal connectivity. This pattern again highlights salience network’s involvement, potentially reflecting a more complex interplay of neural excitability, cue reactivity, and study-specific factors.

In addition, tDCS applied to the left DLPFC has been shown to increase dopamine in the fronto-striatal pathway, part of the brain’s reward system [58,127,128]. This may have therapeutic implications for addictions.

Taken together, these findings indicate that tDCS can systematically modulate salience and reward-related circuits over the DLPFC. By reinforcing the ability of the prefrontal cortex to detect and manage salient drug cues, tDCS appears to reduce the potency of conditioned responses in subcortical reward structures, helping individuals resist habitual triggers and cravings. Over multiple sessions, such neuromodulatory effects may potentiate more enduring structural and functional modifications, ultimately supporting better therapeutic outcomes in addiction treatment.

#### 4.1.2. Single-Session vs. Multiple-Session Effects

A key distinction arises between single-session and multi-session tDCS protocols. Single-session studies—mainly in nicotine [82,84] and methamphetamine use [90,91,92,93]—often document acute changes in neural activation and connectivity without consistently translating into robust shifts in objective clinical measures (e.g., tobacco consumption or complete relapse prevention). Nonetheless, even isolated tDCS sessions can reduce subjective craving [84,90,92], alter default mode network (DMN) and executive control network (ECN) dynamics [82,84,90,92], or modulate frontoparietal connections in response to drug cues [91,93]. This suggests that a single 20–30 min stimulation session can transiently reshape how the brain processes drug-related stimuli, even if the impact on long-term behavior remains limited.

By contrast, multiple-session trials in smoking [83] and alcohol use disorder [86,87,88,89] highlight the potential for accumulating neural and clinical benefits. For instance, while repeated tDCS in smokers did not produce group-level differences in cigarette consumption relative to sham, it did yield enhanced craving reduction and changes in posterior cingulate activation [83]. Similarly, multi-session approaches in alcohol dependence produced more durable modulation of network properties—seen as increased global efficiency in functional connectivity [86] and heightened directed connectivity from the stimulated prefrontal cortex to addiction-related circuits [89]. These findings underscore the principle that repeated tDCS can induce longer-lasting neuroplastic changes (e.g., strengthened white matter integrity [87] or more stable PFC–subcortical coupling [86,89]) that may better support clinical recovery.

#### 4.1.3. Behavioral and Clinical Outcomes

A noteworthy observation is the variability in whether tDCS yielded measurable changes in smoking, craving, or task performance. For instance, a single-session protocol [82] did not significantly alter behavioral measures in smokers during cognitive tasks (N-back, Faces, or Parametric Flanker), even though activation changes were detected in the anterior cingulate cortex (ACC) and default mode network (DMN). Likewise, in repeated-session protocols for smoking [83], cigarette consumption initially decreased across both active and sham tDCS groups, suggesting the effect might owe more to study engagement or expectation. However, there was a distinct advantage in the active group for craving reduction, consistent with the possibility that repeated tDCS—though not affecting consumption—could attenuate subjective urges. In another single-session study [84], real tDCS yielded smaller increases in craving during cue exposure than sham but did not alter task accuracy or reaction times. These findings mirror prior tDCS research in addiction, where craving appears more malleable to stimulation than objective usage measures.

For alcohol use, the results were somewhat more consistent in showing clinically meaningful changes. In one trial [86], multiple sessions of right-anodal/left-cathodal DLPFC tDCS increased the time to first alcohol use after discharge and improved global network efficiency. Another study [88] found that anodal right/cathodal left DLPFC stimulation (five sessions) increased prefrontal responsiveness to alcohol cues and correlated with prolonged abstinence. Still, some single-session or short-term tDCS studies in alcohol dependence have reported less robust behavioral changes; it appears repeated exposures may be necessary to produce clinically relevant effects.

For stimulants such as methamphetamine, two studies [90,92] found that a single or short series of tDCS sessions could reduce craving and alter large-scale connectivity. In contrast, one larger trial [91] did not observe a differential effect on craving compared to sham, despite significant brain activation changes. These discrepant findings may reflect differences in participant characteristics, baseline craving levels, or the exact tDCS montage and intensity.

#### 4.1.4. Task-Based Versus Resting-State Findings

In the tDCS studies summarized, a key distinction emerges between measuring neural activity and connectivity during task-based paradigms versus at rest. Task-based fMRI illuminates how tDCS can acutely modulate brain responses while individuals perform cognitive or cue-reactivity tasks, thereby capturing immediate network engagement under goal-directed conditions. For instance, single-session paradigms in nicotine [82] or go/no-go tasks [85] show that anodal DLPFC stimulation can shift BOLD signal and connectivity in regions like the anterior cingulate cortex (ACC) or parietal cortex, sometimes translating into marginal but notable behavioral benefits such as improved accuracy or reduced craving. Similarly, cue-reactivity studies [84,90,91] indicate that tDCS targeting the DLPFC can recalibrate salience and reward processing networks during exposure to drug cues, with changes in the ACC, insula, and precuneus correlating with reductions in craving, even when objective consumption remains unchanged.

By contrast, resting-state connectivity assessments—seen in studies including [84,86,90,92]—offer insight into the intrinsic organization of large-scale networks like the default mode (DMN) and executive control (ECN) networks following tDCS, independent of any external task. These analyses consistently reveal that tDCS can reconfigure baseline network dynamics, particularly in repeated-session protocols where connectivity alterations (e.g., enhanced global efficiency or decreased DMN hyperconnectivity) correlate with extended time to relapse or reduced craving in alcohol- and stimulant-dependent populations. The suggestion is that offline neural “recalibration” may outlast the stimulation period, potentially reflecting greater plastic changes in the circuitry that modulates craving and self-control.

Integrating task-based and resting-state approaches provides a comprehensive perspective on tDCS’s efficacy. While task-based fMRI highlights real-time effects—such as the attenuation of drug cue reactivity or improvements in working memory performance—resting-state fMRI captures more generalized shifts in network structure that may underpin lasting behavioral improvements, particularly after multiple tDCS sessions. This dual method helps researchers identify the immediate cognitive domains most impacted by stimulation and determine whether these acute changes transition into stable neuroplastic adaptations across extended periods.

#### 4.1.5. Substance-Specific Patterns of tDCS Efficacy and Neural Modulation Based on fMRI Results

Across the fMRI literature, it is clear that the impact of prefrontal tDCS is not uniform but varies systematically with the psychoactive substance, stimulation dose, and neurobiological status of the participants when they receive it.

For nicotine, most experiments deliver only a single 20–30 min session (1–2 mA) to the DLPFC while smokers are still using; the main neural signature is a transient, task-locked modulation—typically a deeper deactivation of default-mode regions such as the precuneus and posterior cingulate during high-load working memory tasks, or a polarity-dependent boost of ACC activity during error monitoring. Behaviorally, these acute neural shifts translate into modest, state-dependent effects: craving rises less steeply during a cue task, but cigarette consumption and carbon monoxide output remain unchanged unless stimulation is repeated over several days.

By contrast, studies on alcohol dependence and stimulant (methamphetamine, crack-cocaine) use disorders employ short courses of bilateral DLPFC stimulation (five to ten 2 mA sessions). In alcohol use disorder, multi-session tDCS reliably reintegrates large-scale networks—global efficiency increases, global clustering decreases, and directed connectivity from the left DLPFC to incentive salience and negative emotion networks is strengthened. These graph-level changes predict clinical benefit: longer stop-signal reaction times improve, time to first drink doubles, and relapse rates fall at one- to four-month follow-up. Methamphetamine studies show a different—but equally robust—pattern: a single right-anode/left-cathode session is enough to rebalance large-scale connectivity, suppressing posterior DMN links while boosting ECN and ventral attention coupling; the magnitude of this network reconfiguration scales with the fall in subjective craving. When five sessions are given, additional plastic changes emerge—white matter integrity in the vmPFC → nucleus-accumbens tract increases, and those microstructural gains track further reductions in stimulant craving.

These substance-specific outcomes can be understood mechanistically. In nicotine dependence, the dominant pathology is an acute frontal hypodopaminergic state during withdrawal; a brief excitatory push to the DLPFC suffices to normalize DMN suppression during cognitive load, but it cannot by itself overcome the ingrained, cue-driven routines that sustain smoking. Alcohol and stimulant disorders, in contrast, are marked by chronically weakened executive control, hyper-salient limbic responses, and, for stimulants, pronounced DMN hyperconnectivity. Repeated stimulation is therefore required to rewrite the broader network architecture—either by re-linking the DLPFC with ACC and salience hubs in alcohol, or by restoring ECN dominance over an overactive DMN in methamphetamine. Dose and montage matter as well: the alcohol trials that achieved the largest behavioral benefits delivered roughly ten milliamp hours of current across five days with a cathode-left/anode-right montage, while the most successful methamphetamine protocols targeted the right DLPFC with the strongest simulated electric fields in fronto-parietal circuits. Finally, stage of abstinence modulates responsiveness: smokers tested while still consuming show smaller gains than alcohol or stimulant users assessed in early detox, a window where fronto-limbic circuits are particularly plastic and their dysconnectivity more severe.

### 4.2. EEG Outcomes

These studies underscore both the promise and the complexity of using tDCS to modulate neural processes related to SUDs. Although methodological details (e.g., electrode montages, target cortical areas, stimulation intensities) differ, a common theme emerges: tDCS can produce measurable changes in electrophysiological indices (e.g., ERP amplitudes, resting-state EEG activity, functional connectivity) that often correlate with cognitive and clinical outcomes such as inhibitory control, craving, or attentional bias. Yet, the observed effects are neither uniform nor immediate. Across the included investigations, several critical points warrant emphasis. The studies demonstrate that tDCS over the DLPFC enhances P3 amplitudes, a marker of attentional resource allocation, particularly in Go/NoGo and cue reactivity tasks [94,95]. Enhanced P3 amplitudes have been linked to improved inhibitory control and reduced attentional bias toward substance-related cues [96]. Furthermore, tDCS has been shown to modulate N2 and ERN responses, which are associated with conflict monitoring and error processing, respectively, indicating a potential mechanism for reducing compulsive drug-seeking behaviors [97,98]. ERP components may serve as functional indices. The observed ERP modulations can be mapped onto a model: the N2 component is tied to ACC-mediated conflict detection, and the P3 is to allocation of attentional resources and inhibitory processing. Our summary of studies suggests that anodal DLPFC tDCS often enhances these components (greater N2/P3 amplitude) during cognitive control tasks. We interpret this as evidence that the prefrontal “control signal” is stronger after stimulation, consistent with increased ECN drive. Changes in late potentials (LPP) to drug cues—often showing reduced amplitude—may reflect diminished affective salience, possibly due to a downregulated limbic response. However, we caution that most ERP findings are correlational and sample-specific; more work is needed to link them causally to network changes.

First, the temporal dimension of tDCS effects appears particularly relevant. Studies incorporating longer follow-ups (e.g., [94]) suggest that immediate post-stimulation assessments may not capture all relevant neural or behavioral shifts. In [94], for instance, no robust post-treatment differences emerged in smokers at the one-day assessment point. Yet, group disparities in reaction times and NoGo-related P3 amplitudes became evident at three months. Similar lags have been noted in alcohol use disorder research, where acute tDCS sessions sometimes fail to produce immediate behavioral changes. Still, subsequent follow-ups reveal more pronounced group differences in either neural markers or clinical outcomes (e.g., [96,100,105]). These findings suggest that plastic changes in neural circuitry may require time—and potentially repeated stimulation—to translate into detectable behavioral improvements. The delayed emergence of neurophysiological changes following tDCS has been reported in multiple studies, suggesting a time-dependent trajectory of neural plasticity. While immediate post-stimulation assessments may show modest ERP changes, longitudinal studies indicate that neurophysiological effects, such as increased NoGo-P3 amplitudes and reduced reaction times, become more pronounced over weeks to months [98,99]. For example, a study on tobacco smokers demonstrated that while ERP responses remained unchanged at 24 h post-stimulation, significant reductions in craving and improved executive function emerged at a three-month follow-up [94]. This aligns with the hypothesis that tDCS-induced neuroadaptations follow a progressive trajectory, requiring sustained intervention to optimize cognitive outcomes.

Second, the location and polarity of stimulation may differentially modulate SUD-relevant cognitive and affective processes. Most studies targeted the bilateral dorsolateral prefrontal cortex (DLPFC) because of its known involvement in inhibitory control, decision making, and craving regulation. Yet, variations in whether the anode was placed on the right or left DLPFC (e.g., [94,101,102]) and whether electrodes targeted alternative frontal regions (e.g., right inferior frontal gyrus, as in [98]) may alter downstream neural responses. For instance, bilateral DLPFC montages were frequently associated with modulations in ERP components linked to attention (P3, LPP) or conflict detection (N2, ERN), mainly when drug-related cues were used. Notably, studies [87,101,104] indicated that bilateral stimulation could differentially affect activation in deeper structures such as the anterior cingulate cortex (ACC) or ventromedial prefrontal cortex (vmPFC), highlighting the potential for tDCS to influence distributed networks rather than only superficial cortical sites.

Third, the heterogeneity of SUD populations and experimental paradigms complicates direct comparisons across studies. Populations ranged from individuals with nicotine dependence [94] to those with alcohol [87,95,96,97,98], opioid [101], amphetamine [100], cocaine, or methamphetamine use disorders [102,103,104]. Each group introduces unique patterns of neural dysfunction and clinical symptomatology, potentially influencing tDCS response. Likewise, differences in cognitive tasks—Go/NoGo tasks [94,99], cue reactivity paradigms [87,102], mindfulness-based interventions [98], and approach bias retraining [95]—target distinct facets of SUD-related behavior. While many studies evaluated P3 and other ERP components during these tasks, the exact manner by which tDCS interacts with cue type, cognitive load, or emotional salience remains an ongoing question. For example, some results [99] suggest that binge drinkers show distinct ERP modulations (e.g., elevated N2 for correct Go trials) under active stimulation. Yet, these changes do not necessarily translate into overt performance improvements. This misalignment between neural and behavioral indices may reflect a “ceiling effect” in well-practiced tasks or imply that early neural changes require further consolidation or more intense therapeutic interventions to manifest as lasting behavioral gains.

Fourth, electrophysiological findings are important mechanistic clues as to how tDCS might work. EEG findings on altered ERP components post-tDCS are further supported by neuroimaging data demonstrating functional connectivity changes in addiction-related circuits. Specifically, individuals who exhibit greater reductions in P3 amplitudes to drug cues following tDCS also demonstrate enhanced functional connectivity between the DLPFC and anterior cingulate cortex (ACC), suggesting improved top–down regulation of craving and impulse control [100,101]. Additionally, EEG-fMRI studies report that the modulation of theta oscillatory activity in the frontal cortex correlates with reduced connectivity within the default mode network (DMN), a network implicated in self-referential processing and craving states [102,103]. These findings highlight the interplay between tDCS-driven neuroplasticity at both local (cortical) and network (large-scale connectivity) levels. Across studies [95,100,101,104,105], changes in P3 amplitudes, N2 latencies, LPP magnitudes, and resting-state connectivity suggest a reorganization of large-scale neuronal networks. Consistently, alterations in frontal–parietal or frontal–temporal connectivity were noted [101,105], implicating an influence of tDCS on cognitive control circuits. In parallel, some studies [87,96] demonstrated that participants showing more significant reductions in craving or more pronounced changes in executive function scores also exhibited distinct ERP or source-localization shifts in key frontal regions (e.g., vmPFC, dorsolateral prefrontal cortex, ACC). Nevertheless, even when neural measures were robustly altered, the correlations with clinical measures of craving, abstinence, or executive functioning were not always linear. This points to the necessity of multimodal approaches (combining electrophysiological, behavioral, and clinical endpoints) and perhaps personalized stimulation protocols to accommodate individual variability in brain network organization.

Finally, interpretational caveats and future directions warrant mention. Sample sizes in many studies were modest, limiting statistical power and generalizability. Although multiple sessions were more common in recent designs, the field still lacks consensus about the “optimal dose” (intensity, frequency, duration) for tDCS in SUD treatment. Furthermore, many participants presented with comorbid conditions (e.g., depression, anxiety, polysubstance use), which may modify neural reactivity to tDCS and complicate group comparisons. Some investigations’ inconclusive or null findings [94,95,98,99] underscore that tDCS effects can be subtle, potentially overshadowed by task constraints, placebo responses, or compensatory cognitive strategies. Notably, the direction of electrophysiological impacts (e.g., increased vs. decreased amplitude in frontal regions) may depend on whether the desired outcome is heightened inhibitory control or reduced sensitivity to drug cues. Hence, future trials should adopt longitudinal designs with extended follow-ups, carefully characterize individual neurobiological profiles, and integrate neuroimaging (e.g., fMRI, PET) with EEG to comprehensively map tDCS-induced network-level changes.

In conclusion, the growing body of evidence suggests that frontal tDCS can modulate electrophysiological indices relevant to craving, inhibitory control, and attention in SUDs—changes that may, under certain conditions, translate to beneficial behavioral or clinical outcomes. However, not all individuals or substance types respond equally, and the time course of emerging effects remains variable. Overall, this is in line with prior work highlighting the utility of EEG-based functional connectivity in assessing central nervous system dynamics in health and disease, as well as EEG applications in diagnostics of other disorders [129,130].

### 4.3. fNIRS Outcomes

Study [106] showed that tDCS applied to the left DLPFC increased the connectivity between the orbitofrontal cortex channel (CH48) and the dorsolateral prefrontal region in tobacco smokers. Functional near-infrared spectroscopy (fNIRS) offers a portable, cost-effective way to capture prefrontal hemodynamics (oxy- and deoxyhemoglobin changes) and functional connectivity before and after tDCS sessions. fNIRS findings shed light on two main mechanisms by which tDCS may shape addicted brain circuits: (1) altering connectivity in executive–reward networks and (2) modulating craving-related circuits.

tDCS may alter functional connectivity in large-scale executive and reward networks. For instance, the DLPFC and orbitofrontal cortex (OFC) are key nodes in the top-down regulation of craving. Studies measuring resting-state connectivity via fNIRS have revealed hyperconnectivity between the left and right OFC in active heroin users, a pattern associated with anxiety and compulsive drug-seeking [131]. When tDCS is delivered to these frontal areas, preliminary data from the study in this review suggest connectivity imbalances can be partly reversed, promoting more typical OFC involvement in decision making and diminishing overactivation linked to craving. The strengthening of executive control networks may correlate with a drop in default-mode or reward-related connectivity, which could be measured in future studies using fNIRS or combined EEG–fNIRS approaches. This remapping toward “task-positive” networks is believed to underlie better impulse inhibition and reduced drug salience.

Finally, the modulation of craving-related networks is a pivotal mechanism by which tDCS drives neuroplastic benefits. Cue reactivity studies consistently show that midline cortical hyperactivity underlies subjective cravings [132]. By facilitating dorsolateral prefrontal and executive circuits, tDCS dampens such hyperactivity. Owing to its high temporal sampling rate, fNIRS can capture these acute tDCS-induced changes during or immediately after stimulation. Over multiple sessions, such acute effects can consolidate into longer-lasting network reconfiguration, consistent with the decreased PFC cue reactivity reported in people who maintain abstinence.

### 4.4. Blood Test Outcomes

In study [107], in addition to lowering the degree of depression, anxiety, stress, and craving, stimulating the DLPFC resulted in a substantial shift in BDNF levels. A member of the neurotrophin family of growth factors, BDNF is crucial for neurons’ survival, growth, and operation. BDNF is produced as proBDNF, which is cleaved into its mature form, mBDNF, and is encoded by the BDNF gene found on human chromosome 11 [133]. To initiate downstream signaling cascades, such as the PI3K/Akt, PLCγ, and MAPK (mitogen-activated protein kinase) pathways, BDNF mainly binds to its high-affinity receptor TrkB (tropomyosin receptor kinase B). These pathways control neuronal survival, differentiation, neurogenesis (the production of new neurons), synaptic plasticity (the strengthening of synaptic connections), and long-term potentiation (LTP), which is essential for memory and learning [134,135]. In the context of addictions, BDNF helps shape long-term adaptations to repeated substance use, influencing both reward pathways and higher-order cognitive functions. Many drugs of abuse (e.g., alcohol, cocaine, opioids) alter BDNF levels and signaling in a region-specific manner. Chronic drug use commonly modifies BDNF expression in the ventral tegmental area (VTA), nucleus accumbens (NAc), and prefrontal cortex. Changes to BDNF within these areas can either exacerbate or dampen drug-seeking behaviors, depending on which neural circuits are affected. For instance, upregulation of BDNF in the medial prefrontal cortex has been linked to reduced addictive-like behaviors, whereas altered BDNF in the VTA or striatum can promote drug craving or relapse [136,137]. In alcohol dependence, several clinical and preclinical studies reveal significant correlations between BDNF expression and drinking patterns, withdrawal severity, and risk of relapse. Rodents that escalate from moderate to excessive alcohol intake often show decreasing BDNF levels in certain corticostriatal areas, whereas restoring BDNF can reduce alcohol self-administration. Similar findings have been reported with psychostimulants such as cocaine, where time-dependent “incubation” of craving has been linked to rising or dysregulated BDNF activity in mesocorticolimbic circuits [138,139]. Individuals actively using alcohol or stimulants frequently present lower peripheral BDNF compared to controls, with partial normalization in abstinence. While peripheral BDNF does not always move in lockstep with CNS levels, multiple studies suggest that its concentration in serum or plasma may track vulnerability to relapse or severity of dependence [140].

Although direct measurements of BDNF before and after tDCS in human addiction populations are still somewhat limited, emerging data show an association between tDCS sessions and changes in peripheral BDNF levels. One hypothesis is that repeated anodal stimulation of the left DLPFC can heighten cortical activity and induce neurotrophin expression in associated neural circuits. This upregulation may stabilize newly formed synapses that promote abstinence-related behaviors. Moreover, by elevating local cortical excitability and promoting beneficial neuroplastic changes, tDCS-induced increases in BDNF could help reorganize or strengthen neural networks that support sustained recovery. For example, higher BDNF levels might bolster connectivity in frontal–striatal circuits, enabling better cognitive control in the face of drug cues and stressors known to trigger relapse.

In study [108], after anodal stimulation of right DLPFC, TNF-α and IL-6 decreased significantly. Despite these decreases, between-group comparisons for cytokine changes did not reach overall significance, partly because IL-6 and TNF-α increased in the sham group. Neuroinflammation in SUDs typically involves a cascade of immune-related events in the central nervous system that reinforce compulsive drug-seeking and relapse. Chronic exposure to substances such as alcohol, cocaine, heroin, and methamphetamine prompts hyperactivation of resident immune cells, namely microglia and astrocytes, in key reward and stress circuitry (including the medial prefrontal cortex, nucleus accumbens, hippocampus, and amygdala). Once activated, these glial cells upregulate the synthesis and release of proinflammatory mediators—particularly TNF-α, IL-1β, and IL-6—along with chemokines and reactive oxygen species. These proinflammatory signals compromise the blood–brain barrier by weakening tight junctions and facilitating leukocyte infiltration into the brain parenchyma. Meanwhile, excessive cytokine release and glial reactivity directly interfere with neuronal functioning. For instance, elevated IL-1β and TNF-α can reduce the expression or function of glutamate transporters (e.g., GLT-1) on astrocytes, increasing extracellular glutamate and potentially leading to excitotoxic damage. Similarly, prolonged microglial activation can alter dopaminergic and GABAergic pathways, skewing neural plasticity in favor of drug-craving circuits [141,142,143,144,145,146].

tDCS applied to the left DLPFC can help counteract these processes by diminishing proinflammatory cytokines such as TNF-α and IL-6. tDCS delivers a mild electrical current to shift neuronal membrane potentials, thus modulating local excitability. By stabilizing activity in the prefrontal cortex, tDCS can reduce excessive neuroimmune activation and, in turn, lower the release of inflammatory mediators. Improved executive function and emotional regulation, combined with this dampening of inflammation, may support recovery by reducing craving and vulnerability to relapse. Although additional research is needed to confirm and refine these findings, current data suggest that interventions aimed at normalizing neuroinflammation—of which tDCS is one promising example—could be a valuable component of treatment strategies for addictive disorders.

## 5. Integrated Neurobiological Model of tDCS in SUDs

Possible mechanisms of action of tDCS in SUDs are already known, derived from numerous neuroimaging and biochemical studies. To collectively summarize these results, a conceptual, integrated neurobiological model of tDCS in SUDs is proposed. This framework shows how the results from the studies included in this review can explain the behavioral improvements often observed in patients with addictions.

### 5.1. Modulation of Prefrontal Circuits and Networks

Anodal tDCS targeting the DLPFC engages a cascade of network-level changes in the addicted brain. Functional MRI studies show that stimulating the DLPFC can strengthen connectivity within the frontoparietal executive control network (ECN) and salience networks (including regions like the ACC and insula), while dampening hyperconnectivity in the default mode network (DMN) [92]. For example, bilateral DLPFC tDCS in methamphetamine users increased coupling among ECN and attention networks and decreased ECN–DMN connectivity, effects that coincided with acute craving reduction [92]. Such findings align with broader observations that people with substance dependence have disrupted large-scale networks—in heroin dependence, baseline FC between ECN, DMN, and salience networks is abnormally low, correlating with poor self-control and high craving [147,148,149,150,151,152]. Neuromodulation helps “bridge the gap”: abstinence or brain stimulation can increase cross-network connectivity, which in turn is consistently associated with lower craving and relapse risk. In essence, tDCS appears to rebalance neurocircuitry: heightening DLPFC-mediated top–down control and normalizing network dynamics (e.g., reducing DMN overactivity) that underlie craving-related maladaptive thoughts [153]. Notably, the ACC—a hub for cognitive control and emotion regulation—is a key node impacted by DLPFC stimulation. One fMRI trial found that 10 sessions of DLPFC tDCS increased resting-state connectivity between the right DLPFC and ACC, and this enhanced DLPFC–ACC coupling correlated with faster stop-signal reaction times (improved response inhibition) in addicts [154]. This suggests that tDCS may restore the interaction between prefrontal “brakes” (DLPFC/ECN) and salience monitoring (ACC), thus strengthening the neural capacity for inhibitory control over drug-seeking impulses.

### 5.2. Craving, Inhibitory Control, and Clinical Outcomes

By modulating these circuits, tDCS can alter the neural processing of drug cues and self-regulation of craving. The DLPFC and ACC together exert top–down regulation over the reward/salience system (including striatal and limbic regions). When DLPFC activity or connectivity is boosted, patients show reduced cue-provoked limbic reactivity and improved executive oversight [155,156]. Clinically, this manifests as dampened craving intensity and fewer lapses. For instance, tDCS during drug cue exposure can reduce self-reported craving immediately, presumably by engaging frontal networks that down-regulate the motivational salience of the cues. Concurrently, enhancing executive network activity helps patients exert self-control in the face of triggers—a change reflected in better task performance (e.g., improved Go/No-Go or stop-signal inhibition after real vs. sham stimulation). Over repeated sessions, these acute effects may translate into tangible clinical benefits such as lower relapse rates. Indeed, strengthening of fronto–striatal and fronto–limbic connectivity through neuromodulation is emerging as a predictor of sustained abstinence. In summary, tDCS likely attenuates the cycle of addiction by simultaneously reducing the “pull” of drug cues (via modulation of DMN and salience circuit activity) and increasing the “pushback” from cognitive control circuits (via DLPFC/ECN and ACC engagement). This dual action addresses two core drivers of relapse: intense cue-induced craving and impaired inhibitory control.

### 5.3. Insights from EEG (ERP) Markers

Changes in event-related potential (ERP) components provide convergent evidence of these neurocognitive effects. Addicted individuals often show blunted N2 and P3 amplitudes during cognitive tasks, indicating deficits in conflict monitoring (N2) [157,158] and attentional resource allocation (P3) [159,160] that may correlate with poor impulse control. Conversely, drug-related cues elicit exaggerated late positive potentials (LPPs) or P3 responses, reflecting attentional bias and motivational salience of the cues [161,162]. Therapeutic neuromodulation can normalize these ERP markers. For example, a single DLPFC tDCS session in cocaine users reduced the N2 amplitude to drug cue Go/NoGo stimuli [102], suggesting that drug cues became less conflict-inducing or attention-grabbing after stimulation. Repeated sessions have been shown to augment P3 amplitudes in target contexts: after five daily tDCS sessions, addicts exhibited a larger P3 to drug cues (particularly originating from frontal regions like vmPFC), compared to sham [104]. This increase in P3 reflects an improved engagement of cognitive–evaluative processes when confronting drug cues, potentially indicating that patients are allocating more attention to task-relevant processing instead of being “hijacked” by the cue’s salience. Notably, anodal left DLPFC tDCS in alcohol dependence was reported to significantly increase the P300 ERP amplitude [97], essentially counteracting the abnormally low P3 seen in addiction and related conditions. Because P300 generation is linked to dopaminergic frontal systems and working memory updating [163,164], its enhancement via tDCS may signal a restoration of dopamine-mediated cognitive function. Similarly, the LPP, which indexes sustained attention to emotional or drug-related stimuli [165,166], has shown promise as a biomarker of cue-reactivity and craving intensity. Early studies and meta-analyses indicate that higher LPP responses to drug cues correlate with greater subjective craving and risk of relapse [167]. By extension, reducing LPP magnitude through interventions (e.g., via improved frontal regulation of attention) could signify a reduction in the motivational grip of drug cues. Although direct evidence of tDCS effects on the LPP is still growing, the observed normalization of P3/N2 suggests that tDCS helps recalibrate addicts’ real-time brain responses, increasing neural signatures of attentional control while diminishing those of cue-induced bias. Importantly, these ERP changes are not merely epiphenomena—they have been linked to behavior. For instance, boosting No-Go P3 amplitude is associated with fewer impulsive errors, and a reduction in cue-triggered potentials corresponds with lower self-reported urge. This reinforces the idea of causal efficacy: tDCS-driven ERP modulations likely mediate improvements in self-control and craving regulation, rather than just paralleling them.

### 5.4. Neuroplastic and Molecular Underpinnings

Beyond these electrophysiological and connectivity changes, tDCS may create a brain environment more conducive to recovery via neuroplastic and neurochemical mechanisms. Brain-derived neurotrophic factor (BDNF), a key protein for synaptic plasticity and neural health, appears to be upregulated by prefrontal tDCC. Clinical studies in SUD and depression report increased serum BDNF levels following repeated DLPFC tDCS. Mechanistically, the tonic depolarization induced by anodal stimulation elevates cortical activity, which in turn triggers activity-dependent BDNF release and TrkB receptor signaling cascades that strengthen synapses. In the context of addiction, where chronic drug exposure and stress can erode synaptic connectivity and reduce BDNF, this tDCS-facilitated boost in neurotrophic support may help renormalize prefrontal–striatal circuits and improve the learning of new, non-addictive behaviors. Parallel to enhancing plasticity, tDCS may also mitigate neurobiological factors that perpetuate addiction pathology, such as neuroinflammation. Sustained substance use is associated with elevated proinflammatory cytokines and microglial activation, which can impair neural function (and even suppress BDNF expression). Notably, emerging evidence (primarily from translational and animal studies) indicates that tDCS exerts anti-inflammatory effects, lowering levels of cytokines like TNF-α and IL-6 and shifting microglia toward a neuroprotective phenotype. By alleviating low-grade neuroinflammation, tDCS might release the brakes on neuroplastic recovery—for example, reducing inflammation can disinhibit BDNF gene transcription, amplifying the neuroplastic gains described above. Therefore, our model posits that tDCS works on multiple timescales: acutely modulating neural circuits and excitability, and more gradually optimizing the brain’s milieu for repair (via BDNF-mediated synaptic strengthening and reduced inflammatory stress). These molecular changes could underpin the durability of clinical improvements, helping to consolidate the acute craving reductions and cognitive boosts into longer-term resilience against relapse.

### 5.5. Conceptual Framework’s Limitations

While this cohesive model is compelling, it is critical to acknowledge the limitations of current evidence. Many of the links in this chain are supported by correlational findings rather than direct proof of causation. For instance, tDCS studies have observed pre–post changes in fMRI connectivity or ERP magnitudes alongside clinical improvements, but it remains inferred that the neural changes drive the symptom changes. We cannot yet definitively say, for example, that increasing a patient’s P300 amplitude is what causes their craving to abate—both could be parallel outcomes of stimulation. Similarly, reports of altered DLPFC–ACC connectivity or DMN deactivation with tDCS are intriguing, but these are often small-sample studies, and replication is needed to ensure reliability. The quality of evidence varies: many trials have modest sample sizes and heterogeneous designs, raising concerns about publication bias and generalizability. There is also an overreliance on proxy biomarkers: increases in BDNF or decreases in cytokines are promising signs of enhanced plasticity, but we lack longitudinal data tying these biomarker shifts to actual relapse outcomes. To strengthen causal claims, future research will need to employ rigorous controls and multimodal approaches, for example, linking tDCS-induced brain changes to behavior in real time, or using predictive biomarkers to see if those who achieve larger network/ERP changes maintain longer sobriety. Additionally, we must consider that the mechanistic picture may differ across individuals and substances. The DLPFC-anchored circuit model largely stems from alcohol and stimulant studies; opioids or other SUDs might involve partly different circuitry or require alternative targeting. In sum, the therapeutic model presented provides a unifying narrative for how tDCS might ameliorate SUD neurobiology, but it should be interpreted with caution. The framework is supported by convergent evidence (fMRI, EEG, and biomarker studies), yet most of this evidence is indirect and based on association. High-quality, causal experiments—perhaps combining tDCS with neuroimaging, neurochemical monitoring, and careful clinical follow-up—are needed to confirm each link. By critically evaluating these limitations, we refine our understanding and avoid overstating tDCS as a panacea. Instead, we recognize it as a promising tool that modulates brain circuits and plasticity in ways consistent with reduced craving and relapse, while committing to further research to firmly establish how and to what extent these neural modulations translate to sustained recovery.

## 6. Limitations and Future Directions

### 6.1. Limitations of Generalizing Across Diverse Studies

Despite a growing body of work linking tDCS to neuro-behavioral change in SUDs, the evidence base remains heterogeneous in ways that complicate cross-study synthesis and the formulation of unified mechanistic claims.

The included trials span nicotine, alcohol, methamphetamine, opioids, cocaine/crack-cocaine, mixed stimulants, and hazardous drinking without a formal diagnosis. Each drug class engages partially overlapping but ultimately distinct neurochemical systems (e.g., cholinergic vs. dopaminergic vs. opioid receptors) and learning histories, which shape both the baseline state of large-scale networks and their plasticity window during tDCS. Attempting to generalize a tDCS-induced change in default-mode network suppression observed in abstinent smokers to, say, cue-elicited anterior cingulate modulation in crack users risks over-interpreting convergent topography as convergent mechanism.

Across studies, stimulation montages (left vs. right DLPFC anodal, bilateral, fronto-occipital, inferior frontal), current intensities (1–2 mA), session numbers (single to ten daily sessions), and online vs. offline task paradigms vary widely. Even nominally “bilateral DLPFC” montages differ in electrode size, spacing, and reference placement, yielding markedly different modeled electric field distributions. Additionally, outcome measures range from fMRI deactivation to EEG ERPs, serum cytokines, and clinical relapse rates. Such methodological scatter limits meta-analytic aggregation and makes mechanistic inference contingent on study-specific design choices.

Most trials enroll < 30 participants per arm, are heavily male-dominant (e.g., 100% male in several stimulant studies), and seldom stratify by sex, hormonal status, or age. Given emerging evidence that cortical excitability, neuroinflammation, and relapse trajectories are sexually dimorphic, the external validity of current findings is restricted.

Participants differ in withdrawal state (acute vs. early abstinence vs. maintenance therapy), comorbid mood/anxiety disorders, and exposure to behavioral interventions such as cognitive bias modification or mindfulness-based relapse prevention. Because many trials permit stable pharmacotherapy or psychosocial treatment, attributing neural or clinical change exclusively to tDCS is precarious.

While a few studies extend the follow-up to three months or four months, most post-assessments occur within hours or days of the final session, leaving questions open about durability and dose–response trajectories. Moreover, mechanistic readouts (e.g., network efficiency shifts) are rarely linked to long-term clinical endpoints across substances.

Progress over the next decade will hinge on designing studies that treat each drug class as its own neurobiological ecosystem—one whose circuitry, neurochemistry, and relapse dynamics place boundary conditions on how prefrontal tDCS can exert influence. First, trialists should develop parallel, substance-stratified protocols in which montage, current density, session schedule, and behavioral context are identical within—yet deliberately distinct across—nicotine, alcohol, opioid, stimulant, and polysubstance cohorts. Only then can researchers compare “apples to apples” within a drug class before cautiously generalizing outward.

Second, harmonizing outcome batteries is imperative. A minimal mechanistic toolkit could pair individualized head modeling through electric field strength with a core set of imaging and electrophysiological readouts—resting-state fMRI for network topology, task-based EEG for millisecond-scale excitability, and ecological sampling of craving and affect. Administering these measures on a shared timeline (immediate, one month, three months, six months) will allow investigators to trace dose–response curves and durability across substances rather than relying on single-time-point snapshots.

Third, precision electric field modeling should move from post hoc explanation to prospective stratification. Predicted cortical dose—derived from each participant’s MRI—can serve as an inclusion threshold, a randomization stratum, or even an adaptive dosing variable, ensuring that “active” stimulation truly engages the target circuit across heterogeneous skull and cerebrospinal fluid geometries.

Fourth, adequately powered, multi-site RCTs must replace the current landscape of under-sized, single-site experiments. Enrolling at least 100 participants per substance and balancing for sex and age will not only stabilize effect-size estimates but also illuminate biologically plausible moderators—such as hormonal status, inflammatory tone, or genetic polymorphisms in neuromodulatory receptors—that may dictate responsiveness to tDCS.

Fifth, factorial designs that embed tDCS within established pharmacological and behavioral treatments are needed to disentangle additive from synergistic effects. For example, combining prefrontal stimulation with buprenorphine maintenance, varenicline, or cue-exposure therapy could reveal whether tDCS principally enhances extinction learning, reduces withdrawal-related dysphoria, or modulates executive control, and whether these mechanisms differ by substance.

### 6.2. Methodological Limitations That Constrain Interpretation—And Practical Steps to Overcome Them

In the current research on tDCS for SUD, electrode montages vary from bifrontal to fronto-occipital, current densities span a two-fold range, and experiments run anywhere from a single exposure to ten twice-daily sessions. Because electric field topographies shift sharply with each of these parameters, it is rarely safe to assume that two “active” protocols are tapping the same cortical circuitry. The field therefore needs a tiered standardization strategy. At a minimum, all new trials should (i) publish their exact electrode geometry and individualized head-model dose maps in open repositories, and (ii) anchor their designs to one of a small set of consensus “reference” montages so that genuine replications can accumulate. Large consortia can then experiment systematically—varying only one element (e.g., session number) while holding the rest constant—to build dose–response curves instead of one-off case studies.

Small, demographically skewed samples impose a second, equally stringent ceiling on inference. Most stimulant and opioid studies remain > 90% male, and the median per-arm sample hovers below thirty—numbers that inflate effect sizes and preclude sex-by-stimulation moderation tests. The remedy is collective, rather than individual: multi-site RCTs powered for at least moderate effects (n ≈ 100 per substance) and stratified by sex, age, and hormonal status. Harmonized screening, shared data-capture platforms, and centralized randomization will make these larger trials feasible without untenable cost inflation.

A third obstacle lies in inconsistent sham protocols and weak blinding checks. Ramp-up/ramp-down, low-dose continuous currents, or shoulder montages are all labeled “sham,” yet scalp sensations and participant expectancies differ markedly among them. Future studies should adopt internationally vetted sham waveforms whose sensory profile has been psychophysically matched to the target montage, and they should report formal blinding-integrity statistics (e.g., Bang indices). Where expectations remain a concern, balanced-placebo designs or active-control montages that mimic tingling but direct current away from prefrontal targets can further isolate neurobiological effects.

Finally, analytic flexibility erodes confidence in positive findings. With dozens of neural, behavioral, and biochemical endpoints—and few adjustments for multiple testing—the risk of chance discovery is high. Mandatory pre-registration of primary and secondary outcomes, along with publicly shared code and raw datasets, will curb this problem. Journals and funders can accelerate the shift by prioritizing registered report formats and by awarding replication grants that explicitly repeat high-value protocols in independent laboratories.

### 6.3. Translational and Clinical Limitations—And Strategic Routes Forward

The promise of prefrontal tDCS in addiction neuroscience rests on a chain of evidence that still shows several weak links when translated from laboratory signals to lasting clinical benefit. First, the field leans heavily on surrogate neuro-markers—deactivation of the default-mode network, shifts in ERP amplitudes, graph-theoretic gains in efficiency—without consistently demonstrating that these neural shifts forecast harder endpoints such as biologically verified abstinence, delay to first lapse, or cumulative consumption. A straightforward remedy is to embed those behavioral outcomes as co-primary measures in every study and to model explicitly, via mediation analysis, whether the neural change explains a significant slice of variance in relapse risk; only the biomarkers that survive this test should be promoted as genuine mechanistic targets.

A second limitation is temporal: most follow-ups end within days of the final stimulation, leaving the durability of both neural and behavioral effects essentially unknown. Requiring outcome assessments at one, three, six, and twelve months—made feasible through inexpensive ecological-momentary-assessment apps and postal biomarker kits—would allow investigators to chart dose–response curves and construct Kaplan–Meier survival functions that can be compared across trials.

Third, the ecological validity of prevailing laboratory cue reactivity tasks is modest. Stock photographs or sounds delivered in a scanner seldom evoke the multisensory, socially embedded cues that precipitate real-world relapse. Integrating geofenced smartphone prompts and wearable physiology (e.g., heart-rate variability, skin conductance) can capture autonomic and craving responses during naturally occurring triggers, providing a bridge between controlled and everyday environments.

Fourth, concurrent pharmacotherapy and psychosocial treatment introduce uncontrolled moderators; it remains unclear whether tDCS adds, multiplies, or merely duplicates their effects. Factorial and SMART trial designs that cross tDCS with standard care will clarify interaction patterns, while the meticulous reporting of medication doses and therapy hours will let future meta-analysts stratify accordingly.

Fifth, individual variability—stemming from skull morphology, baseline network topology, systemic inflammation, or genetic polymorphisms—means that a fixed 2 mA protocol will under-dose some patients and overshoot others. Collecting baseline MRI, EEG, C-reactive protein, cytokine panels, and polygenic risk scores, and then using Bayesian adaptive algorithms to titrate current until a prespecified electric field threshold or neurophysiological signature is reached, would move the field toward precision dosing.

### 6.4. Uncertainty About the Most Effective Stimulation Parameters

The studies included in this review most often used anodal stimulation of the left DLPCF, and less frequently the right. The only certain conclusion is that stimulation of these areas has complex neurobiological effects. Numerous review studies examining the efficacy of tDCS in treating SUDs also used anodal stimulation of the left and right DLPFC. Many of them showed benefits in reducing cravings, reducing relapse frequency, etc. However, in all studies, including those using neuroimaging techniques, there was a wide variety of treatment protocols. Differences occurred in the assembly of reference electrodes, intensity used, stimulation frequency, and total number of stimulation sessions. Some studies in this review showed that a single stimulation session changed brain activity. This shows that a single session is capable of inducing a neurobiological effect. However, tDCS is often studied in the context of relieving symptoms of various psychiatric and neurological diseases using high-intensity protocols, e.g., 10 sessions, daily on weekdays for 2 weeks. We speculate that a higher number of sessions may yield greater results in the treatment of addictions. In terms of current intensity, the most commonly used current intensity is 1.5–2 mA. These intensities are consistent with most studies using behavioral measures to measure the effects of tDCS in addictions. Taking these observations together, it is conservative to conclude that stimulation of the left DLPCF in particular, at 1.5–2 mA, and with longer treatment protocols (>5 sessions), may yield greater neurobiological and behavioral effects. However, a challenge for future researchers is to examine different tDCS protocols in the context of inducing neurobiological changes. Will there be a correlation between an increased number of stimulations and greater effects seen in brain scans and biochemical measures, which would translate into greater behavioral outcomes?

## 7. Conclusions

This review indicates that transcranial direct current stimulation can induce measurable neurobiological changes in individuals with substance use disorders. Neuroimaging studies consistently show that tDCS modulates activity in large-scale brain networks—particularly when targeting the dorsolateral prefrontal cortex—with effects correlating to reduced craving and improved inhibitory control. Electrophysiological measures (e.g., P3, LPP) and blood biomarkers (e.g., BDNF, IL-6, TNF-α) also show promising modulation following repeated stimulation. However, variability in protocols and limited long-term data remain key challenges. Future research should focus on standardization, identifying mediators of clinical efficacy, and clarifying which patients benefit most from tDCS.

## Figures and Tables

**Table 1:** Studies using fMRI in SUDs.

Notable Observations	Key Neural and Behavioral Findings	Tasks/Measures	tDCS Protocol	Sample and Design	Study
- Despite no overt behavioral effect, there was a pronounced **tDCS-induced DMN deactivation** under the left-DLPFC-anode montage. - ACC activity changes emerged specifically for correct Flanker responses.	- **Behavior**: No significant main or interaction effects of tDCS; nicotine-abstinent smokers performed worse/slower across tasks. - **ROI Flanker**: tDCS × Group interaction in R ACC (*p* = 0.03), greatest under An-lDLPFC in sated smokers. - **Whole-brain N-back**: Stronger DMN deactivation under An-lDLPFC vs. reversed polarity or sham (*p* < 0.01). - **Nicotine sated**: Additional DMN deactivation in 3-back minus 0-back.	- Modified Faces/Shapes (amygdala reactivity). - Parametric Flanker (ACC/insular activity). - N-back (WM, DMN suppression). - fMRI during or shortly after tDCS.	- Single-session tDCS but repeated in 3 separate conditions (left anodal DLPFC/right cathodal vmPFC, reversed polarity, sham). - 2 mA, 25 min each.	- 15 smokers (7F), 28 nonsmokers (14F). - All underwent 3 tDCS sessions (double-blind, crossover). - Smokers tested once while nicotine-sated (patch), once while abstinent (placebo patch, ≥12 h).	[82]
- **Craving** was notably reduced by active tDCS, despite no difference in actual cigarette consumption or CO levels. - A dorsal PCC cluster showed **opposite** activation changes in active vs. sham.	- **Smoking**: Consumption decreased over 5 days then rose after; **no** between-group difference. - **Craving**: Active group had stronger session-by-session craving reduction (*p* = 0.031). - **fMRI**: A right dorsal posterior cingulate region showed time × group interaction; activity ↑ in active group, ↓ in sham (*p* < 0.005 uncorrected). - No correlation with smoking behavior changes.	- Daily cigarette use, exhaled CO. - Self-reported craving pre-/post-session. - fMRI cue reactivity (smoking, neutral, target images) pre- vs. post-tDCS.	- **Active** vs. **Sham** (anode ~F4–Fp2 over right DLPFC, cathode left occipital). - 2 mA, 20 min per session.	- 29 adult smokers wanting to quit. - Randomized, double-blind, parallel arms. - 10 sessions over 5 days (2×/day).	[83]
- Demonstrates a **direct link** between fronto-hippocampal connectivity and craving modulation. -Single 30 min session was sufficient to alter neural reactivity to smoking cues.	- **Craving**: Overall rose from pre- to post-cue, but **smaller increase** under real vs. sham (*p* = 0.027). - **Cue reactivity fMRI**: tDCS × Cue interaction in L superior and middle frontal gyri; smoking-minus-neutral activation was reduced more with real tDCS (*p* < 0.01). - **Connectivity**: PPI between L DLPFC and R parahippocampal gyrus was altered by real tDCS (*p* < 0.001), correlating with craving changes (r = −0.52). - No effect on emotion task or reaction times.	- Pre/post-craving ratings. - Resting-state fMRI. - Smoking cue reactivity (neutral vs. smoking pics). - Emotion-processing task (negative vs. neutral).	- Anode over left DLPFC, cathode over right DLPFC. - 1 mA, 30 min. - tDCS administered inside MRI scanner.	- 32 male chronic smokers (≥10 cig/day, ≥2 y). - Within-subject, counterbalanced (real vs. sham). - 1-week interval. - 2 tDCS sessions–real and sham.	[84]
- Indicates **lateralized** effects: anodal right DLPFC speeds up go responses; anodal left DLPFC modifies other regions’ activity/connectivity. - Single session can alter network-level BOLD, even without large behavioral shifts on No-Go.	- **Behavior**: Right-DLPFC group had faster Go RT vs. Sham (*p* = 0.008). No-go errors unchanged. - **BOLD**: Both left and right groups had decreased activation in occipital/precuneus areas for Go. Right group had increased STG/cerebellar activation in No-Go. - **Connectivity**: Post-stimulation changes in DLPFC-based networks, with distinct patterns for left vs. right anode. - No direct craving measure reported.	- Go/No-Go performance (RT, errors) - BOLD signal changes pre- vs. Post-stimulation - Seed-based connectivity (left or right DLPFC)	- 2 mA, 20 min. - Cathode on contralateral supraorbital area. - Pre/post fMRI during Go/No-Go.	- 46 right-handed male smokers. - Randomly assigned to left anode DLPFC, right anode DLPFC, or sham. - Single tDCS session.	[85]
- Suggests **fronto-cingulate network reorganization** can improve impulse control and prolong abstinence. - Graph metrics (efficiency/clustering) predicted relapse risk.	**Connectivity**: Active group ↑ global efficiency (*p* = 0.007) and ↓ global clustering (*p* = 0.008); sham no change. - Sub-network analysis found stronger connectivity in right ACC and frontal nodes. **Clinical**: Active group took ~30 days to relapse vs. ~15 for Sham (*p* = 0.013). - Greater reductions in global clustering correlated with faster stop-signal response times (r = 0.57).	- Resting-state fMRI (pre/post). - Graph–theoretic network measures (global efficiency/clustering). - Time to first alcohol use after discharge.	- Anode on right DLPFC (F4), cathode on left DLPFC (F3). - 2 mA, 20 min.	- 24 males with alcohol use disorder. -Randomized double-blind to **active or sham.** - 5 sessions (once/day).	[86]
- Evidence that bilateral DLPFC tDCS can **structurally strengthen** specific fronto-limbic connections. - White matter changes closely tracked **craving improvements**.	- Only the **left vmPFC–NAcc** white matter tract changed significantly in active tDCS (↑ voxel count, FA, ADC, all *p* < 0.05). - Gains in left vmPFC–NAcc DTI indices correlated with **greater craving reduction** (R^2^ ≥ 0.29, *p* < 0.05). - No changes in other tracts.	- ERP for cue reactivity. - DTI focusing on 3 tracts: VTA–NAcc, VTA–PFC, vmPFC–NAcc. - Craving scales.	- Cathode on left DLPFC (F3), anode on right DLPFC (F4). - Typically 2 mA, 20 min or 13 + 20 + 13 min protocols.	- 14 men (7 active, 7 sham), with alcohol or crack-cocaine dependence. - 5 sessions. - Behavioral, EEG/ERP, DTI measures.	[87]
- Even a short 5-day course can **upregulate frontal responsiveness** to alcohol cues, tied to reduced relapse. - Supports **frontal “boost”** in controlling cue-related urges.	- At baseline, no group difference in BOLD reactivity. Post-tDCS, **real** group had **increased DLPFC activation** to alcohol cues vs. sham. - This DLPFC increase correlated with **longer abstinence** (*p* = 0.048).	- Baseline and post-treatment fMRI with a visual alcohol cue paradigm (VICE). - Time to lapse after treatment.	- 2 mA, 20 min - Cathode over left DLPFC, anode over right DLPFC.	- 24 patients with alcohol dependence after detox. - Real vs. sham tDCS, daily for 5 days.	[88]
- Provides evidence that strengthening top–down control to subcortical/limbic networks fosters **reduced relapse**. - **Directed** connectivity measures highlight a possible **causal** effect of tDCS.	**Connectivity**: Active group ↑ directed connections from lDLPFC to incentive salience and negative emotionality networks, sham group ↓. - This connectivity gain predicted staying abstinent at 4 months. - Relapse rate: 19% active vs. 38% sham; difference more pronounced among females.	- Resting-state fMRI (pre/post). - “Causal discovery” methods of connectivity in 4 hypothesized addiction networks. - Relapse monitoring at 1 and 4 months.	- Anode on left DLPFC (F3), cathode on right DLPFC (F4). - 2 mA, 20 min/day.	- 60 AUD patients in early abstinence (residential). - Randomized to active vs. sham. - 5 days of tDCS + cognitive training.	[89]
- Demonstrates that **bilateral DLPFC** stimulation can **reconfigure** network connectivity in early meth abstinence and yield immediate craving relief.	- **Craving**: Active tDCS → ~15-point drop, sham → ~1-point drop (*p* = 0.03). - **Connectivity**: Decreases in DMN (posterior cingulate, precuneus), increases in ECN (parietal, temporal), and SN (lingual gyrus, cingulate). - Larger connectivity shifts correlated with bigger craving reductions.	- Resting-state fMRI (pre/post). - Self-reported meth craving (0–100). - Large-scale network connectivity (DMN, ECN, SN).	- Anode on right DLPFC (F4), cathode on left DLPFC (F3). - 2 mA, 20 min each session.	- 15 male participants in early abstinence from methamphetamine. - Double-blind, crossover (active vs. sham). - 1-week washout. - 2 session of tDCS (real and sham).	[90]
- Shows that actual electric field (EF) strength in targeted cortical sites predicts local functional changes. - No net difference in craving, but **distinct BOLD trajectories** for active vs. sham.	- **Craving**: Both groups declined, no group difference. - **fMRI**: sham → decreased activation on 2nd exposure; active → increased BOLD in L MFG, insula, IPL, precuneus, IFG. - EF in right SFG correlated with local BOLD and frontoparietal connectivity changes (only in active).	- fMRI cue reactivity (meth vs. neutral images). - Computational head models to map EF distribution. - Self-reported craving.	- Anode on right DLPFC, cathode contralateral supraorbital. - 2 mA, 20 min.	- 60 adult males in residential treatment for methamphetamine use. - Randomized, single-session (active vs. sham). - Triple-blind design. - Single tDCS session.	[91]
- Suggests rebalancing among large-scale networks (ECN, VAN, DMN) that parallels craving reduction. - Head-model EF intensities correlated with these connectivity shifts.	- **Craving**: active → larger drop vs. sham. - **gPPI**: From an ECN seed, connectivity with visual/precuneus region ↑ in active, but ↓ in sham. - DMN seed connectivity with a parietal cluster ↓ in active, ↑ in sham. - Gains in ECN/VAN connectivity, reduced DMN connectivity.	- fMRI during pictorial meth cue reactivity task. - Seeds in ECN, DMN, VAN. - Craving measured pre/post.	- Bilateral DLPFC: anode at F4, cathode at F3. - 2 mA, 20 min.	- 15 men with MUD in residential program. - Double-blind, crossover (active vs. sham). - 1-week washout. - 2 tDCS sessions (real and sham).	[92]
- Highlights that tDCS “dose” (EF intensity) may shape **network** connectivity rather than localized activation. - No significant group-level craving benefit from single session.	- No robust EF–fMRI correlations at voxel/ROI/cluster levels (corrected). - **Network-level** analysis: EF in right-frontal regions positively correlated with frontoparietal connectivity changes (r = 0.43, *p* = 0.03). - Craving did not differ significantly real vs. sham.	- Structural MRI to build head-model Efs. - fMRI cue reactivity (meth vs. neutral) pre-/post. - Craving assessments.	- Anode over right DLPFC (F4), cathode over left eyebrow (Fp1). - 2 mA, 20 min.	- 60 males with MUD. - Randomized to real vs. sham. - Single session in MRI scanner.	[93]

Abbreviations: DLPFC: dorsolateral prefrontal cortex; vmPFC: ventromedial prefrontal cortex; ACC: anterior cingulate cortex; l/r: left/right STG/MFG/SFG: superior/middle frontal gyrus; DMN: default mode network; ECN: executive control network; SN/VAN: salience network/ventral attention network; VTA: ventral tegmental area; NAcc: nucleus accumbens; FA/ADC: fractional anisotropy/apparent diffusion coefficient (DTI metrics); BOLD: blood-oxygen-level dependent (fMRI); gPPI: generalized psychophysiological interaction (connectivity analysis).

**Table 2:** Studies using EEG in SUDs.

Notable Observations	Key Findings	Tasks/Measures	tDCS Protocol	Sample and Design	Study
- Suggests **delayed** tDCS effects on inhibitory control - P3 amplitude change tied to attenuated salience of smoking cues	- **1-day post:** No between-group differences in RT, accuracy, or ERPs - **3-month post**: Active tDCS → faster RT (esp. post-correct) and reduced NoGo P3 for smoking images vs. sham	- Go–NoGo task with smoking vs. neutral images - EEG: N2, P3, ERN amplitudes	- Bilateral DLPFC: anode R-DLPFC (F4), cathode L-DLPFC (F3)-2 mA - 13 min ×2 (with 20 min break), per session	- 73 daily tobacco smokers - 6 total sessions over 3 days, plus 3 lab visits (pre, 1-day post, 3-month FU)	[94]
- Minimal synergy between tDCS and CBM on behavior - EEG indicated **some** craving benefit with active tDCS - Overall, changes in approach bias not driven by tDCS	- **CBM** alone reduced approach bias but effect mainly in 1st session - **tDCS** had no main effect on bias or consumption - In cue reactivity EEG, an interaction (time × tDCS) showed active tDCS → greater reduction in craving for alcohol images post-assessment	- Approach Avoidance Task (AAT) - Implicit Association Test (IAT) - Self-reported craving and alcohol use - EEG (oddball and cue-reactivity tasks; P300)	- Left DLPFC montage - 1 mA, 15 min - Cathode contralateral supraorbital	- 78 hazardous drinkers (AUDIT > 8) - 2 × 2 design (sham/active tDCS × Control/Active CBM) - 3 training sessions	[95]
- Contrasting results: real tDCS lowered craving and depression but had higher relapse rate - Points to complexity of clinical vs. electrophysiological outcomes	- **Relapse**: 66.7% real vs. 14.3% sham (*p* = 0.053) - **Craving** decreased more under real tDCS (Δ = −9.1 vs. −1.5, *p* = 0.015) - **Depression** reduced more (*p* = 0.005) - ERP changes were smaller in real tDCS group vs. bigger shifts in sham	- Craving, depression, anxiety, QoL - Executive functioning (Frontal Assessment Battery) - ERPs (neutral/alcohol pics), LORETA analysis	- Anode on left DLPFC - Cathode on contralateral (right) supradeltoid - 2 mA, 20 min, 1×/week ×5	- 13 male Lesch Type IV alcoholics - Weekly “real” anodal tDCS vs. “sham” for 5 consecutive weeks	[96]
- Effects are strongly **subtype dependent** (Type IV responded most positively) - Highlights **individual difference** in tDCS response	- Alcohol-related sounds → ↑ P3 amplitude post–tDCS, stronger in **real** vs. sham - **Type IV**: greatest P3 rise at all electrode sites + improved FAB with real tDCS - **Type II**: P3 amplitude decreased under real vs. sham - No changes in craving	- P3 amplitude to alcohol vs. neutral sounds - Frontal Assessment Battery, daily alcohol consumption by subtype	- Left DLPFC, 1 mA, 10 min - Cathode on contralateral supradeltoid - Focus on P3 amplitude at Fz, Cz, Pz	- 49 outpatients with alcohol dependence (Lesch Types I–IV) - 2 sessions (sham vs. real) - ERP recordings (before/during/after)	[97]
- Suggests **vmPFC** involvement is crucial for maintaining abstinence w/bilateral tDCS - LORETA localized distinct cortical generators of P3 changes	- Active, abstinent participants showed largest P3 changes in **vmPFC** - Sham/relapse participants had shifts in middle temporal gyrus - A similar pattern emerged for crack-cocaine subset	- P3 amplitude changes w/drug images - Brain areas (vmPFC vs. Others) - Abstinence vs. relapse - LORETA	- Bilateral DLPFC: cathode L-DLPFC, anode rightDLPFC - 2 mA, 20 min	- 22 men (8 active, 14 sham) -alcohol or crack-cocaine dependence - 5 tDCS sessions	[87]
- Suggests MBRP is key driver of craving and LPP changes - Active tDCS had no robust incremental effect - LPP correlates w/emotional cue processing	- Overall craving and LPP to alcohol decreased significantly pre→post (*p* < 0.02) - **tDCS** did **not** significantly improve craving vs. sham - Higher baseline LPP in the active group - More MBRP sessions → greater craving reduction	- EEG w/alcohol, neutral, negative images - Late positive potential (LPP) - Self-reported craving and negativity ratings	- tDCS over right IFG (F10), 2 mA, 20 min - Cathode on left upper arm	- 68 participants (alcohol use disorder/heavy drinking) - 8-session MBRP group + active vs. sham tDCS	[98]
- tDCS modulated early conflict (N2) in binge drinkers - Non-binge drinkers had stronger inhibitory P3 w/tDCS - Illustrates **subgroup differences** in neural responsiveness	- Behavioral performance similar across conditions and groups - **Binge**: Larger N2 amplitude under active, no P3 change - **Non-binge**: Larger No-Go P3 under active vs. sham - No direct correlation between ERP and performance	- Behavioral (RTs, No-Go accuracy) - ERPs: N2, P3 for correct Go/No-Go	- Left DLPFC, 1.5 mA - Cathode above right eye (Fp2) - tDCS applied during a neutral Go/No-Go	- 40 participants (20 binge, 20 non-binge) - Within-subject, 2 sessions (active vs. sham), 1-week apart	[99]
- Suggests **enhanced attentional control** for neutral stimuli - Real tDCS uniquely improved invalid-cue performance over 5 days	- Only real tDCS group improved on invalid-minus-valid RT - P300 amplitude (300–410 ms) increased in real tDCS group under neutral cues - No drug-cue P300 changes - Sham/control → no improvement	- Posner cueing task (drug/neutral) measuring attentional RT - EEG P300 for invalid vs. valid targets	- Cathode on left DLPFC (F3), anode on R-DLPFC (F4) - 2 mA, 13:20:13 protocol, daily ×5	- 30 total (10 real tDCS, 9 sham, 11 control) - Abstinent amphetamine users vs. controls - 5-day regimen of tDCS	[100]
- Suggests repeated bilateral tDCS can **reorganize** resting-state EEG, especially connectivity in slower frequencies. - No direct craving or usage measure.	- **Active** (both directions): Larger reductions in slow-wave amplitude (delta–alpha) than sham. - Greater increases in coherence (delta, theta, beta) in frontal/parietal/temporal. - Sham had smaller or different patterns.	- EEG amplitude (delta, theta, alpha, beta) - EEG coherence (connectivity) among 19 channels	- 2 mA, 20 min, bilateral DLPFC	- 30 men w/opioid use disorder on MMT - 10 daily sessions of tDCS vs. sham - 3 groups: left-anode/right-cathode, right-anode/left-cathode, sham	[101]
- Single bilateral session can **dampen ACC** reactivity to crack cues. - Sham shows typical “rise” in ACC under drug stimuli.	- Sham: ACC current density **increased** from baseline for crack images. - Active: ACC density **decreased** for crack images (*p* < 0.0001). Neutral unaffected.	- EEG (N2 ~200–350 ms) - Crack vs. neutral images - LORETA in ACC	- 2 mA, 20 min - Anodal right DLPFC, cathodal left DLPFC	- 13 adults with crack-cocaine addiction - Left-cathode/right-anode vs. sham - Single session	[102]
- Suggests a single left-cathodal/right-anodal session can **blunt** left DLPFC reactivity - Reinforces the role of DLPFC in cue-provoked responding	- **Real** tDCS prevented typical rise in left DLPFC activation when viewing crack images (*p* < 0.0001) - Sham group did not show this suppression; no changes in right DLPFC - Strong correlation of DLPFC activity with days of abstinence	- Cue reactivity paradigm (drug vs. neutral) - 32-channel EEG + LORETA for DLPFC (BA 9, 46)	- 2 mA, 20 min - Anodal right DLPFC, cathodal left DLPFC	- 16 participants, crack-cocaine dependent (≤30 days abstinent) - Left-cathode/right-anode vs. sham - Single session, then P3 analysis	[103]
- Demonstrates **both immediate and cumulative** tDCS effects on P3 sources in left DLPFC and beyond - Multiple sessions **broaden** the affected prefrontal regions (frontopolar, OFC, ACC)	- After single active session → P3 in L DLPFC ↑ for neutral cues, ↓ for crack cues - Sham → opposite direction - After 5 active sessions → changes extended to frontopolar, orbitofrontal, ACC - Sham → smaller or no changes	- EEG P3 (350–600 ms) for neutral vs. drug cues - LORETA of prefrontal regions	- 2 mA, 20 min - Anodal right DLPFC, cathodal left DLPFC	- 13 crack-cocaine–dependent adults - Randomized to real (left cathode/right anode) vs. sham - 5 successive sessions	[104]
- Single bilateral session specifically reduced **initial attentional bias** (P3) to MA cues - Relationship to network efficiency suggests **baseline connectivity** modulates tDCS response	- **P3**: Real tDCS → drop in amplitude for MA cues vs. sham (*p* = 0.002 at post); LPP unaffected - **Craving**: Dropped more in tDCS group but between-group difference n.s. - **Network topology**: Those with higher local/global efficiency at baseline showed smaller P3 declines	- ERP P3 and LPP to MA vs. neutral - Self-reported craving - Weighted phase lag index and graph efficiency metrics	- 2 mA, anode right DLPFC (F4), cathode left DLPFC (F3)	- 42 men with methamphetamine use disorder, abstinent 1 wk–6 mos - Single bilateral (R-anode/L-cathode) vs. sham	[105]

Abbreviations: DLPFC: dorsolateral prefrontal cortex; vmPFC: ventromedial prefrontal cortex; IFG: inferior frontal gyrus; ACC: anterior cingulate cortex; EEG/ERP: electroencephalogram/event-related potential; P3/N2/ERN/LPP: ERP components (cognitive control; error processing; attention); LORETA: low-resolution electromagnetic tomography; CBM: cognitive bias modification; AAT: Approach Avoidance Task; IAT: Implicit Association Test; FU: follow-up; RT: reaction time; MMT: methadone maintenance therapy.

**Table 3:** Studies using fNIRS in SUDs.

Notable Observations	Key Findings	Tasks/Measures	tDCS Protocol	Sample and Design	Study
- No group differences for **subjective craving** or **LF-HRV** across the session. - **No changes** in high-frequency HRV or LF/HF ratio. - **Greater prefrontal connectivity** (fNIRS) observed with real tDCS, suggesting localized neuromodulatory effects without overtly reducing craving or HRV.	- **Craving** increased significantly over time for both groups (*p* < 0.001), with **no interaction** by tDCS. - **LF-HRV** rose over time (*p* = 0.001), but **no difference** between real vs. sham. - **fNIRS**: Time-by-stimulation effect in left BA9 (*p* = 0.039). Higher **connectivity** between orbitofrontal (CH48) and dorsolateral PFC region (CH6) in real tDCS vs. sham (*d* = 0.66, *p* < 0.001).	- 20 min “in vivo” smoking-cue exposure. - Craving (self-report ratings). - Heart-rate variability (HRV) from ECG (low-frequency, high-frequency, LF/HF ratio). - fNIRS (prefrontal hemodynamics; changes in deoxygenated hemoglobin).	- 2 mA, 115 min. - Anode at F3 (left DLPFC), cathode at Fp2 (orbitofrontal).	- **29** adult smokers (university students smoking at least weekly). - Random assignment to **real** (anode left DLPFC, cathode orbitofrontal) or **sham** tDCS. - Single-session design, with in vivo smoking-cue exposure.	[106]

**Table 4:** Studies using blood tests in SUDs.

Notable Observations	Key Findings	Tasks/Measures	tDCS Protocol	Sample and Design	Study
- Both active tDCS groups had some advantage over sham on specific measures - **No direct difference** emerged between left-anode vs. right-anode groups - Indicates **selective benefits** (e.g., left-anode for mood/stress, right-anode for BDNF)	- **BDNF**: Right-anode group vs. sham was significant (*p* = 0.042); left-anode vs. sham not significant - **Depression**: Left-anode vs. sham (*p* = 0.023) - **Anxiety**: Left-anode vs. sham (*p* = 0.001), right-anode vs. sham (*p* = 0.006) - **Stress**: Left-anode vs. Sham (*p* = 0.014); right-anode vs. sham not significant - **Craving**: Both active protocols significantly differed from sham	- Serum BDNF via ELISA - Depression, anxiety, stress via DASS - Craving via Desires for Drug Questionnaire (DDQ)	- 2 mA for 20 min - Left-anode group: Anode at F3, cathode at F4 - Right-anode group: Anode at F4, cathode at F3	- **30** male patients with opioid use disorder, randomized into 3 groups (n = 10 each): (1) Left-anode/right-cathode DLPFC (2) Right-anode/left-cathode DLPFC (3) Sham	[107]
- Despite **within-group** cytokine reductions in the right-anode group, **between-group** comparisons were nonsignificant - **Sham group** also showed a decrease in craving, though to a lesser extent - The large effect sizes suggest strong impact on craving in both active groups	- **Craving**: Significant overall group effect (ANOVA *p* < 0.001); both active groups had large effect sizes (d > 2.0); sham had a moderate effect (*d* = 0.52) - **Cytokines**: No significant group-level changes (ANOVA *p* > 0.25), but in the right-anode group alone, IL-6 and TNF-α decreased significantly (within-group *p* < 0.01)	- Craving: Desires for Drug Questionnaire (DDQ) - Cytokines (IL-6, TNF-α) via ELISA - Impulsivity: Barratt Impulsiveness Scale (BIS-11)	- 2 mA for 20 min over - bilateral DLPFC (F3 and F4) - Left-anode or right-anode	- **31** men with opioid use disorder, assigned to (1) Left-anode/right-cathode DLPFC (2) Right-anode/left-cathode DLPFC (3) Sham - **Randomized, double-blind, sham-controlled** - 10 consecutive tDCS sessions	[108]

## Data Availability

No new data were created or analyzed in this study. Data sharing is not applicable to this article.
